# Inducing transient enantiomeric excess in a molecular quantum racemic mixture with microwave fields

**DOI:** 10.1038/s41467-023-36653-3

**Published:** 2023-02-20

**Authors:** Wenhao Sun, Denis S. Tikhonov, Himanshi Singh, Amanda L. Steber, Cristóbal Pérez, Melanie Schnell

**Affiliations:** 1 Deutsches Elektronen-Synchrotron DESY, Notkestr. 85, 22607 Hamburg, Germany; 2 Institute of Physical Chemistry, Christian-Albrechts-Universität zu Kiel, Max-Eyth-Str. 1, 24118 Kiel, Germany; 3 Present Address: Departamento de Química Física y Química Inorgánica, Facultad de Ciencias-I.U. CINQUIMA, Universidad de Valladolid, E-47011 Valladolid, Spain

**Keywords:** Chemical physics, Atomic and molecular interactions with photons

## Abstract

Chiral molecules with low enantiomer interconversion barriers racemize even at cryogenic temperatures due to quantum tunneling, forming a racemic mixture that is impossible to separate using conventional chemical methods. Here we both experimentally and theoretically demonstrate a method to create and probe a state-specific enantiomeric enrichment for such molecular systems. The coherent, non-linear, and resonant approach is based on a microwave six-wave mixing scheme and consists of five phase-controlled microwave pulses. The first three pulses induce a chiral wavepacket in a chosen rotational state, while the consecutive two pulses induce a polarization for a particular rotational transition (listen transition) with a magnitude proportional to the enantiomeric excess created. The experiments are performed with the transiently chiral molecule benzyl alcohol, where a chiral molecular response was successfully obtained. This signal demonstrates that enantiomeric excess can be induced in a quantum racemic mixture of a transiently chiral molecule using the developed microwave six-wave mixing scheme, which is an important step towards controlling non-rigid chiral molecular systems.

## Abstract (web summary)

There is growing interest in controlling and manipulating molecules using external field. Here the authors demonstrate microwave induced transient enantiomeric excess in a state-selective benzyl alcohol using microwave six-wave mixing.

## Introduction

Chirality is an ubiquitous and fundamental phenomenon in nature. A chiral molecule has a pair of enantiomers, which are non-superimposable mirror images of each other. They share almost all physical properties, but often possess distinct functionality in chemical reactions and biological activity with chiral environments. For instance, enantiomers of chiral drugs often exhibit different effectiveness and toxicity^1^. As molecular chirality plays an essential role in many fundamental aspects of physics, chemistry, and biology, great efforts have been made to characterize, differentiate, and manipulate chiral molecules. Besides the traditional methods in chemistry, such as chromatography^2^ and capillary electrophoresis^3^, various chiroptical techniques using circularly polarized radiation have been developed to determine the absolute configuration and enantiomeric excess (ee) of a chiral sample, such as vibrational circular dichroism (VCD)^4,5^ and photoelectron circular dichroism (PECD)^6,7^. Recently, the microwave three-wave mixing (M3WM) approach, which is a resonant, coherent, and non-linear spectroscopic technique, became a new methodology for chiral differentiation and quantification^8,9^. The chiral sensitivity arises from the mirror symmetry of the electronic structures of enantiomers, making the triple products of their three electric dipole-moment components (***μ***_*a*_ ⋅ [***μ***_*b*_ × ***μ***_*c*_]) equal in magnitude but opposite in sign, which can be interrogated using a combination of selected rotational transitions.

**Fig. 1:**
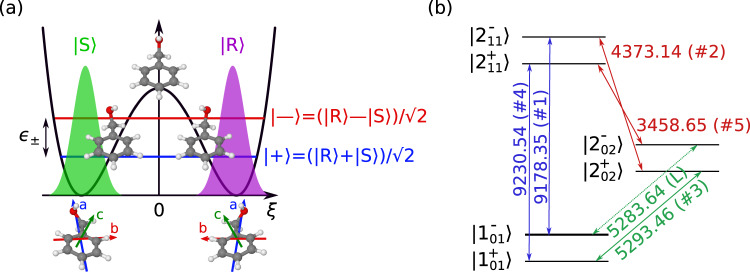
Double-well potential and rotational level scheme. **a** Schematic representation of the enantiomer interconversion mechanism in benzyl alcohol (BA). The $\left|\pm \right\rangle$ states denote the vibronic ground state with opposite parity. The $\left|R\right\rangle$ and $\left|S\right\rangle$ states represent the localized wavepackets of a particular chirality. *ξ* is the large amplitude motion coordinate. *ϵ*_±_ corresponds to the energy difference between the two tunneling states, which is experimentally determined to be 492.816(2) MHz for BA arising from a barrier with a height of about 280 cm^−1^^21^. **b** Rotational level scheme of BA used in our experiment. Each rotational level is marked as ${J}_{{K}_{a}{K}_{c}}^{\pm }$, where *J* is the total angular momentum, *K*_*a*_ and *K*_*c*_ are the projections of the angular momentum onto the molecular axes a and c, and ± indicates the parity of the torsional state. *M* sublevels of the rotational states are omitted here for clarity^29^. The arrows indicate the transitions involved. Each transition is labeled with its frequency (in MHz) and its order in the sequence of the pulses (in parentheses). “L” indicates the “listen” transition, which is not directly excited by the pulse scheme but coherently induced.

In addition to chiral analysis, the M3WM technique showcased its capability to achieve chiral separation at the molecular level^10–13^. Through the interference between the chiral sensitive M3WM path and a direct excitation path, enantiomers can be selectively enriched in chosen rotational states. As the sample is studied in the gas phase with cold molecular sources (e.g. buffer-gas cooling and supersonic jets), this technique can be applied not only to molecules with permanent chirality, such as 1,2-propanediol^10^ and carvone^11^ but also to molecules that exhibit transient chirality, such as cyclohexylmethanol (CHM)^12^, which are impossible to separate via conventional separation techniques, such as chromatography. However, when it comes to molecules that can interconvert their handedness via quantum tunneling through a large-amplitude motion (LAM), the enantiomers can no longer be isolated in a cold supersonic jet, where the molecules are cooled to a rotational temperature (*T*_rot_) of a few Kelvin^14^. The sample naturally appears as a racemic mixture during the jet expansion. These molecules have two (or more) minima on the potential energy surface. Each minimum corresponds to a certain enantiomer, separated by an achiral transition state (Fig. 1). The stationary states of such molecules are of a specific parity with respect to the inversion operation that we will denote as $\left|\pm \right\rangle$. They are the positive and negative combinations of the non-stationary localized wavepackets with a chosen parity ($\left|\pm \right\rangle=\frac{1}{\sqrt{2}}(\left|R\right\rangle \pm \left|S\right\rangle )$). A specific enantiomeric state of such a molecule thus can be achieved by generating a coherent superposition of the states $\left|\pm \right\rangle$ with opposite parity^15–18^. Such an approach has been proposed for rotational states of asymmetric rotors^19,20^; however, to date, no experimental demonstration has been reported.

Herein, we present both theoretical and experimental demonstration of inducing enantiomeric excess from a racemic mixture of a transiently chiral molecule, benzyl alcohol (BA), in the gas phase. This molecule undergoes a LAM, corresponding to a concerted rotation of the CH_2_OH and OH groups in the plane above the phenyl ring. This tunneling motion with a barrier of about 280 cm^−1^ transfers one enantiomer into the other one (see Fig. 1)^21^. It changes the sign of the electric dipole-moment component along the b-axis in the inertial principal axis system (PAS). The corresponding b-type rotational transitions occur between the two tunneling states of opposite symmetry. Because of these interstate transitions, both microwave three-wave mixing^8,22–24^ and the related microwave population transfer schemes become impossible^10,11,22^, as three rotational transitions cannot form a closed cycle due to symmetry considerations. Instead, five resonant excitation pulses are needed to induce the chiral response in the form of a “listen” transition in a suitable system of six rotational levels (see Fig. 1). This chiral response is not directly excited, and its amplitude is dependent on the enantiomeric excess of the sample. By detecting a listen (L) signal with non-zero intensity, we demonstrate that this microwave six-wave mixing (M6WM) scheme is feasible to create a transiently chiral ensemble of BA molecules in the rotational state of interest, despite a fast interconversion of the two enantiomers via the tunneling motion. In the following, we first provide the theoretical derivation of the M6WM scheme, followed by the experimental implementation and demonstration.

## Results

### Principle of the M6WM approach

We will start with an introduction of the M6WM scheme, which consists of five sequential resonant pulses. They correspond to a closed loop of six transitions that connect six states (${\, J}_{{K}_{a}{K}_{c}}^{\pm }$) in the following sequence: $\left|{1}_{01}^{-}\right\rangle \to \left|{2}_{11}^{-}\right\rangle \to \left|{2}_{02}^{+}\right\rangle \to \left|{1}_{01}^{+}\right\rangle \to \left|{2}_{11}^{+}\right\rangle \to \left|{2}_{02}^{-}\right\rangle$, as illustrated in Fig. 1. Here, ${J}_{{K}_{a}{K}_{c}}$ denote the asymmetric rotor rotational state with *J* being the total angular momentum quantum number, *K*_*a*_/*K*_*c*_ being the projections of this momentum onto the a-/c- principal axes of the molecule, respectively, and ± indicating the parity of the torsional state of the molecule. For a theoretical description, the sequence of pulses is treated as a set of subsequent two-level Rabi oscillations (a detailed derivation is given in the Supplementary Information). After applying the five excitation pulses, a macroscopic polarization **P**, which oscillates with amplitude *A* at the frequency of the $\left|{2}_{02}^{-}\right\rangle \to \left|{1}_{01}^{-}\right\rangle$ listen transition (*ν*_L_), is coherently induced:

1 $${{{{{\bf{P}}}}}}_{{{2}^{-}_{02}} \to {1}_{01}^{-}}\propto {\overbrace{-{{{{{\boldsymbol{\mu }}}}}}_{{{2}^{-}_{02}} \to {{1}^{-}_{01}}} \cdot \sin ({\Omega}_{1}{\tau }_{1}) \cdot \left(\mathop{\prod }\limits_{k=2}^{5} \sin \left(\frac{{\Omega}_{k}{\tau }_{k}}{2}\right)\right)}^{\begin{array}{c}\propto A\end{array}}}\cdot \sin \left(2\pi {\nu }_{{{{{\rm{L}}}}}}t-{\Phi}+\mathop{\sum }\limits_{k=1}^{5}{s}_{k}{\varphi }_{k}\right).$$

where ${{{{{{{{\boldsymbol{\mu }}}}}}}}}_{{{2}^{-}_{02}}\to {1}_{01}^{-}}$ is the transition dipole moment for the induced listen transition $\left|{2}_{02}^{-}\right\rangle \to \left|{1}_{01}^{-}\right\rangle$, ${{{\Omega }}}_{{{{{{{k}}}}}}}=\frac{{{{{{{{{\boldsymbol{\mu }}}}}}}}}_{{{{{{{k}}}}}}}{{{{{{{{\bf{E}}}}}}}}}_{{{{{{{k}}}}}}}}{\hslash }$ is the Rabi frequency of the *k*-th pulse in the M6WM sequence, where the vector **E**_*k*_ describes its field strength and polarization direction, and ***μ***_*k*_ denotes the transition dipole moment of the transition induced with it^25,26^, *τ*_*k*_ and *φ*_*k*_ are the pulse duration and phase, and Φ is the starting phase of the listen signal.

Equation (1) demonstrates the optimal conditions for the observation of the coherently induced polarization ${{{{{{{{\bf{P}}}}}}}}}_{{{2}^{-}_{02}}\to {1}_{01}^{-}}$, the amplitude of which depends on the Rabi frequency. The first pulse is resonant to the $\left|{1}_{01}^{-}\right\rangle \leftrightarrow \left|{2}_{11}^{-}\right\rangle$ intrastate transition. This pulse should be a *π*/2-pulse (*Ω*_1_*τ*_1_ = *π*/2), which drives the population difference between the $\left|{1}_{01}^{-}\right\rangle$ and $\left|{2}_{11}^{-}\right\rangle$ rotational states into coherence. The other four pulses transfer this coherence to the final state $\left|{2}_{02}^{-}\right\rangle$ via the pathway $\left|{2}_{11}^{-}\right\rangle \to \left|{2}_{02}^{+}\right\rangle \to \left|{1}_{01}^{+}\right\rangle \to \left|{2}_{11}^{+}\right\rangle \to \left|{2}_{02}^{-}\right\rangle$, and they should be *π*-pulses (*Ω*_*k*_*τ*_*k*_ = *π* for *k* = 2, …, 5). As a result, a final coherence at the listen transition is obtained with a maximum amplitude (*A*), when *Ω*_*k*_*τ*_k_ equals to *π*/2 for pulse #1, and *π* for pulse #2 – #5. The directions of the transition dipole moments (a-/b-/c-types are mutually orthogonal) also provide the required polarization directions of the pulses. There are three groups of pulses that need to be orthogonal to each other: pulses #1 and #4, pulses #2 and #5, and pulse #3, which is also indicated by the color scheme in Fig. 1b. The latter pulse (#3) should have the same polarization as the listen transition that is being collected.

The absolute phase of ${{{{{{{{\bf{P}}}}}}}}}_{{{2}^{-}_{02}}\to {1}_{01}^{-}}$ depends on specific aspects of the experimental setup, such as the durations and phases of the five excitation pulses, delays between them, and cable lengths^27^. When the phase of pulse #*k* (*φ*_*k*_) is varied, the phase of ${{{{{{{{\bf{P}}}}}}}}}_{{{2}^{-}_{02}}\to {1}_{01}^{-}}$ changes accordingly, with a linear factor *s*_*k*_ = ± 1. The factor *s*_*k*_ equals +1, when the transition goes up in energy (*k* = 1, 4), or equals −1, when the transition goes down in energy (*k* = 2, 3, 5), as described in more detail in the Supplementary Information.

### M6WM spectroscopy

**Fig. 2:**
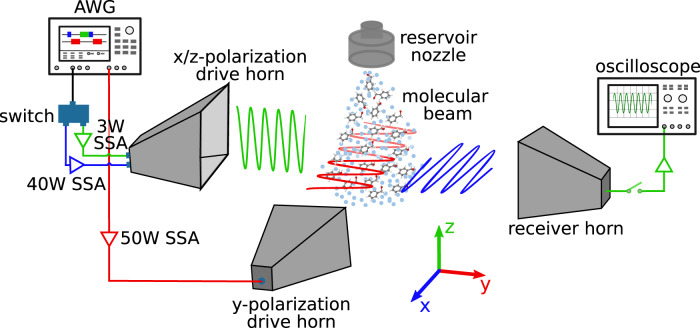
Schematic diagram of the state-specific enantiomeric enrichment experiment. The BA molecular sample seeded in neon with a stagnation pressure of about 3 bar is supersonically expanded into a vacuum chamber via a solenoid valve pulsing at 6 Hz. Five excitation pulses are generated from the two channels of the arbitrary waveform generator (AWG). The pulse sequence from channel 1 (CH1) is fed through the designated outputs of a single-pole-double-throw (SPDT) switch. Blue, red, and green colors represent the polarization directions of the microwave fields in the laboratory frame, which match the color code in the rotational level scheme as provided in Fig. 1. Each branch is equipped with a solid-state amplifier (SSA). The amplified microwave pulses are sequentially broadcast in the designated polarization directions to interact with the molecular jet using two dual-polarization horn antennae. The free induction decay (FID) of the resulting molecular response is collected in the z-polarization direction by a receiver horn and averaged by a fast digital oscilloscope.

The experiment was carried out using a modified broadband Fourier transform microwave spectrometer, as shown schematically in Fig. 2. The molecular sample of BA was introduced into the vacuum chamber via supersonic jet expansions using neon as carrier gas. In the jet, the molecules were rapidly cooled to a *T*_rot_ of ~1–2 K via BA-neon collisions so that a substantial population difference was generated in the rotational states of interest. The ensemble of molecules was sequentially polarized with the five microwave pulses. In order to spatially broadcast the pulses in three mutually orthogonal planes, two dual-polarization horn antennae were installed at 90^∘^ in the vacuum chamber. After polarization, the decay of the induced macroscopic polarization at *ν*_L_ was received by a third horn on the detection side, digitized, and averaged on a fast digital oscilloscope.

**Fig. 3:**
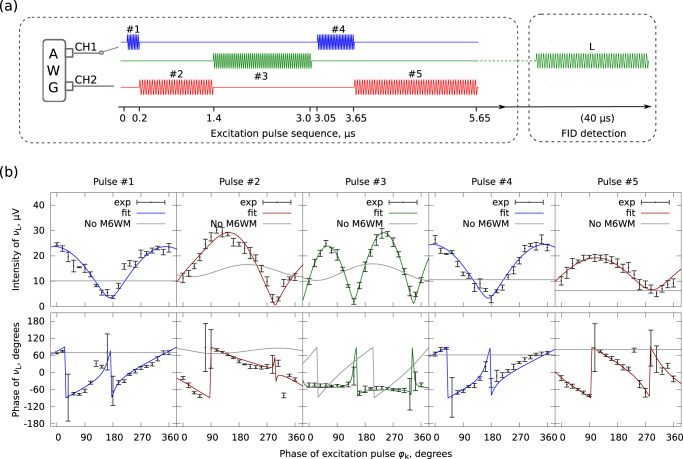
M6WM spectroscopy of BA. **a** The experimental M6WM pulse sequence, where the colors of the microwave pulses match the laboratory frame as in Fig. 2. The durations correspond to an optimized *π*/2-pulse for pulse #1 and *π*-pulses for pulses #2 – #5. 40 μs of FID for the frequency of the listen transition (*ν*_L_) was recorded after the five excitation pulses, the duration of which is not to scale. **b** The resulting phase dependence patterns in amplitude (top) and phase (bottom) at the listen transition (*ν*_L_). They were obtained by individually scanning the phases of the corresponding pulses (*φ*_*k*_) and measuring the respective amplitude and phase of *ν*_L_ at 5283.64 MHz. Here, “phase of *ν*_L_” denotes the arctangent of the tangent of the phase. The experimental results are represented in black with error bars. The errors are calculated from a set of five repeated measurements, in which each phase point was an average of 20,000 FIDs. In the least-squares fits, the amplitudes and the phases (in tangent) are fitted simultaneously. The overall fit for each set is represented in the color that matches the polarization of the corresponding microwave pulse whose phase was varied in the experiment. The gray line in each panel is the simulated amplitude or phase by excluding the M6WM component from the complete fit as discussed in the main text.

Prior to the M6WM experiments, the pulse durations for the *π*/2 or *π* conditions were determined for each pulse by measuring the corresponding nutation curve. The collected nutation curves are provided in the Supplementary Section 2.2, and the optimized pulse sequence is presented in Fig. 3a. The determined pulse durations are approximate to the effective *π*/2 or *π* conditions averaged over *M* subsets of the rotational states^28–30^. Afterwards, we performed a series of M6WM experiments, in which the phase of one excitation pulse (*φ*_*k*_) was systematically varied at a time in steps of 18^∘^ from 0^∘^ to 360^∘^ to evaluate the phase behavior of the M6WM signal at *ν*_L_. The other remaining four pulses were kept unchanged in the scans. At each phase being scanned, 20,000 FIDs were collected and averaged at the frequency of the listen transition, *ν*_L_ = 5283.64 MHz. Each scan was carried out repeatedly five times, allowing us to assess the repeatability of the observations. The results are summarized in Fig. 3b.

### Analysis of the M6WM signal

According to Eq. (1), the molecular response induced from the M6WM cycle, *S*_M6WM_, at the frequency *ν*_L_ of the listen transition ${2}_{02}^{-}-{1}_{01}^{-}$, should have a constant amplitude (*A*_M6WM_) over the five sets of scans, and its phase (*φ*_M6WM_) should be correlated with the phases of the five pulses. In each scan, the phase of one pulse (*φ*) was varied from 0^∘^ to 360^∘^, whereas the phase of the other four pulses remained fixed. The induced polarizations can, thus, be expressed as ${A}_{{{{{{{{\rm{M}}}}}}}}6{{{{{{{\rm{WM}}}}}}}}}\sin (2\pi {\nu }_{{{{{{{{\rm{L}}}}}}}}}t-{{{\Phi }}}^{{\prime} }+{s}_{k}{\varphi }_{k})$, where ${{{\Phi }}}^{{\prime} }$ combines the starting phase Φ in Eq. (1) and the phases of the phase-invariant pulses in the pulse sequence. Hence, *S*_M6WM_ should have a linear phase dependence on *φ*_*k*_ with the coefficient *s*_*k*_. In scans #1 and #4, *s*_*k*_ = +1; in scans #2, #3, and #5, *s*_*k*_ = −1. However, as shown in Fig. 3b, the observations in the experiments exhibit unanticipated phase dependence patterns. The signal intensities at *ν*_L_ are not constant, and the phases are not linearly correlated with *φ*_*k*_.

With careful examinations that are described in the Supplementary Section 2.4, we analyzed that the observed signal at *ν*_L_ was a result of the interference between the expected *S*_M6WM_ signal and perturbation signals at *ν*_L_ (*S*_c2_ and *S*_c3_) when applying pulses #2 and #3. The presence of *S*_c2_ and *S*_c3_ is presumably attributed to off-resonance excitations due to the power leakage in the electronic circuit. The two perturbation signals had constant amplitudes (*A*_c2_ and *A*_c3_) throughout the experiments, and their phases (*φ*_c2_ and *φ*_c3_) were positively and linearly dependent on the phase of pulses #2 and #3, respectively, as shown in Supplementary Figs. 18 and 19 in the Supplementary Information. The phase dependence coefficients of *φ*_c2_ and *φ*_c3_ are denoted as *s*_c2_ and *s*_c3_. In scan #2, where the phase of pulse #2 (*φ*_2_) was scanned, *φ*_c2_ positively and linearly varied with *φ*_2_, while *φ*_c3_ was independent of *φ*_2_. Therefore, *s*_c2_ = + 1, and *s*_c3_ = 0. Likewise, *φ*_c3_ changed along with *φ*_3_ in scan #3 and *s*_c3_ = + 1, whereas *φ*_c2_ did not (*s*_c2_ = 0). In the other three scans, both *φ*_c2_ and *φ*_c3_ remained constant (*s*_c2_ = 0, and *s*_c3_ = 0), as *φ*_2_ and *φ*_3_ were not varied. With a clear understanding of the different phase dependencies of *φ*_c2_, *φ*_c3_, and *φ*_M6WM_, we were able to perform a least-squares fit on each set of the scan to decompose the observed pattern and extract the *S*_M6WM_ signal. The molecular response from each scan is fitted independently as a sum of the individual interfering signals, where the total listen signal amplitude (*A*) and the total phase (Φ) are given by the following expressions:

2 $$\left\{\begin{array}{l}{A}^{2}=\mathop{\sum }\nolimits_{m=1}^{N}\mathop{\sum }\nolimits_{n=1}^{N}{A}_{m}{A}_{n}\cos ({s}_{m}{\varphi }_{k}+{\phi }_{m}-{s}_{n}{\varphi }_{k}-{\phi }_{n})\quad \\ \tan ({{\Phi }})=\frac{\mathop{\sum }\nolimits_{m=1}^{N}{A}_{m}\sin ({s}_{m}{\varphi }_{k}+{\phi }_{m})}{\mathop{\sum }\nolimits_{m=1}^{N}{A}_{m}\cos ({s}_{m}{\varphi }_{k}+{\phi }_{m})}\quad\quad\quad\quad\quad\quad\quad\quad\quad\quad\quad \end{array}\right.\,.$$

Here, *N* is the number of interfering signals, *φ*_*k*_ is the scanned phase, *A*_*m*_, *A*_*n*_, *ϕ*_*m*_, and *ϕ*_*n*_ are the amplitudes and the starting phases of the individual components, which are fitted to the observed experimental signals, *s*_*m*_ and *s*_*n*_ are the associated phase dependence coefficients. The square of the listen signal amplitude (*A*^2^) and the tangent of the listen signal phase ($\tan ({{\Phi }})$) are fitted simultaneously. The details on the procedure and the scripts used for the fitting are given in the Supplementary Information.

**Fig. 4:**
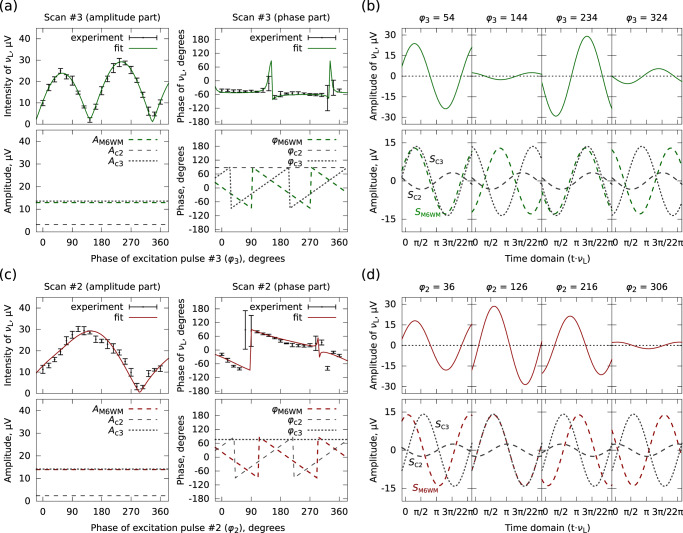
Illustration of the interference patterns. Left: experimentally observed interference patterns and the simulations based on the least-squares fits, when scanning the phase of pulse #3 (**a**) and pulse #2 (**c**). The upper traces show the experimental results together with the overall fit with regard to the intensity and phase at the frequency of the listen transition *ν*_L_, respectively. The errors are calculated from a set of five repeated measurements, as mentioned for Fig. 3. The amplitudes and phases of the individual interfering wave components are presented in the lower traces. In each subplot of the phase part, the phase on the y-axis denotes the arctangent of the tangent of the phase. Right: interpretations of the interference in the time domain at four specific phases of *φ*_3_ (**b**) and of *φ*_2_ (**d**). The upper traces in panels **b** and **d** show the total listen signal in the time domain with the observed amplitude and phase, whereas the lower traces exhibit the three time-domain wave components on the basis of the least-squares fit and the experimental pattern. *S*_M6WM_ (colored dashed line) represents the signal induced from the M6WM scheme. *S*_c2_ (black dashed lines) and *S*_c3_ (black dotted lines) are the co-excited signals together with pulses #2 and #3, respectively.

As in scans #2 and #3, the phases of all three interfering components have different phase dependencies on *φ*_*k*_ (i.e., in scan #2: *s*_c2_ = + 1, *s*_c3_ = 0, and *s*_2_ = − 1; in scan #3: *s*_c2_ = 0, *s*_c3_ = + 1, and *s*_3_ = − 1), they can be explicitly separated in the fit, as shown in Fig. 4. However, in the other three scans (#1, #4, and #5), as both *S*_c2_ and *S*_c3_ are phase-invariant, these two co-excited signals cannot be meaningfully separated. The sum of them is fitted as one phase-invariant signal instead. In each scan, the least-squares fit is obtained over the five repeats, and the experimental uncertainty is derived at each phase point as well. The fit results are summarized in Table 1 and are presented in Fig. 3b together with the experimental observations. The signal component with the anticipated phase dependence is assigned to *S*_M6WM_, and it has a consistently constant amplitude of 13–14 μV throughout the five scans, clearly demonstrating the feasibility of the approach. In Fig. 3b, the curves based on the complete least-squares fits are represented using colored lines, while the simulations of the sum of *S*_c2_ and *S*_c3_ are plotted with gray lines, where the M6WM components are excluded. In this manner, the signal patterns with and without the presence of *S*_M6WM_ are revealed, and detailed demonstrations are further provided in Fig. 4.

**Table 1:** Pulse sequence parameters and the fit results of phase scans

Pulse parameters					*S*_M6WM_		*S*_c2_		*S*_c3_	
#	*ν*, MHz	*τ*, *μ*s	Ω ⋅ *τ*	Type	*s*_M6WM_	*A*_M6WM_, *μ*V	*s*_c2_	*A*_c2_, *μ*V	*s*_c3_	*A*_c3_, *μ*V
1	9178	0.2	$\frac{\pi }{2}$	*c*	+1	14	–	–	0	10
2	4373	1.2	*π*	*b*	−1	14	+1	2	0	14
3	5293	1.6	*π*	*a*	−1	13	0	3	+1	14
4	9231	0.6	*π*	*c*	+1	14	–	–	0	11
5	3459	2.0	*π*	*b*	−1	13	–	–	0	7

"#” denotes the pulse number for which the phase is being scanned, *ν* is the frequency of the pulse, *τ* is the pulse duration, and “type” shows the type of the rotational transition excited by the pulse, as well as the direction of the pulse polarization in the laboratory frame. The *s* are the dimensionless phase dependence coefficients, and the fitted signal amplitudes are denoted by *A*_M6WM_, *A*_c2_, and *A*_c3_, where the subscripts indicate the source of the *ν*_L_ signal. Details on the fitting procedures are in the Supplementary Information. Note that in scans #1, #4, and #5 the sum of *S*_c2_ and *S*_c3_ are fitted, and the corresponding results are shown in the *S*_c3_ columns.

## Discussion

By analyzing the observed interference patterns, we have shown that a signal with a non-zero amplitude at the listen transition (*ν*_L_) can be attributed to our phase-controlled M6WM excitation scheme. The amplitude of this signal (*S*_M6WM_) is constant (13–14 μV) and the phase of it (*φ*_M6WM_) is dependent on the phase *φ*_*k*_ of the *k*-th pulse with the linear factor *s*_*k*_. For scans #2 and #3, all three interfering components (*S*_M6WM_, *S*_c2_, and *S*_c3_) are explicitly disentangled, and the fitting results of the two scans show good agreement. As *φ*_3_ or *φ*_2_ is rotated through 360^∘^, the amplitudes of these wave components stay constant, and their phases exhibit different dependencies on *φ*_3_ or *φ*_2_, which are presented in the lower traces of Fig. 4a, c. In scan #3, *S*_M6WM_ and *S*_c3_ have similar amplitudes, but their phases rotate with *φ*_3_ in opposite directions. As a result, there are two in-phase and two out-of-phase interference coincidences, as demonstrated with the four time-domain snapshots in Fig. 4b, leading to two maxima/minima in the experimentally observed intensity profile (see Fig. 4a). The two unequal minima and maxima are attributed to the interference with the phase-fixed *S*_c2_ component. In contrast, in scan #2, the phase of *S*_c3_ is independent of *φ*_2_, while the phase of *S*_M6WM_ varies with *φ*_2_. This results in only one in-phase and out-of-phase interference for the *S*_M6WM_ and *S*_c3_ signals, one minimum and maximum in the experimental intensity profile, as shown in Fig. 4c and d, respectively. The presence of *S*_c2_ makes the intensity profile slightly asymmetric.

To understand why the signal *S*_M6WM_ observed in the experiment is a sign of induced chiral dynamics, we need to compute the *ee* obtained during the pulse sequence. The signed *ee* is an observable that can be expressed via the following operator^16–18^:

3 $$\hat{ee}=\left|R\right\rangle \left\langle R\right|-\left|S\right\rangle \left\langle S\right |=\left |+\right\rangle \left\langle -\right |+\left|-\right\rangle \left\langle+\right|\,.$$

The pulse sequence and the experiment itself resemble the population transfer experiment followed by a M3WM cycle as a probe of the induced enantiomeric excess as we performed earlier on cyclohexylmethanol (CHM)^12^. For a discussion of the results obtained in the current work, it is useful to compare it with the observations on CHM, where CHM is racemic at room temperature prior to the study, and transient chirality is stabilized in the supersonic jet expansion. The barrier between the enantiomers of CHM is high enough (~15 kJ/mol) to quench the tunneling interconversion of the enantiomers within the time scale of the experiment. Therefore, both the *R* and *S* forms of CHM are present in equal amounts in a gas jet and cannot be converted to each other, leading to ∣*ee*∣ = 0 as a starting condition. In the experiments on CHM, we first performed a population transfer for a set of rotational states, creating a non-zero ∣*ee*∣ in two particular rotational states. This ∣*ee*∣ was then probed by performing an additional M3WM experiment starting from these rotational states.

According to this analogy, in the present study with BA, the first three pulses of the M6WM sequence are analogous to the population transfer part of the experiment, which should induce a non-zero ∣*ee*∣ in a specific rotational state. The last two pulses and the induced coherence for the listen transition act as a chiral probe of this enantiomeric enrichment. To elucidate this, one needs to calculate the ∣*ee*∣ in the system in the rotational state $\left|{1}_{01}\right\rangle$ after three pulses starting, naturally, with a racemic sample. This quantity is given by the operator ${\hat{ee}}_{{1}_{01}}=\left|{1}_{01}^{+}\right\rangle \left\langle {1}_{01}^{-}\right |+\left|{1}_{01}^{-}\right\rangle \left\langle {1}_{01}^{+}\right|$. The explicit calculation (see Supplementary Information for details) shows that after the first three pulses, the resulting state $\left|\psi \right\rangle$ will be an oscillating chiral wavepacket, with an amplitude proportional to ∣*ee*∣:

4 $$\left\langle \psi \right|{\hat{ee}}_{{1}_{01}}\left|\psi \right\rangle \propto {\overbrace{\sin ({{{\Omega }}}_{1}{\tau }_{1})\left(\mathop{\prod }\limits_{k=2}^{3}\sin \left(\frac{{{{\Omega }}}_{k}{\tau }_{k}}{2}\right)\right)}^{\begin{array}{c}\propto|ee|\end{array}}}\cdot \sin \left(2\pi {\nu }_{\pm }t+\mathop{\sum }\limits_{k=1}^{3}{s}_{k}{\varphi }_{k}-{{{\Phi }}}^{{\prime} }\right).$$

The frequency of the *R* ↔ *S* interconversion is simply a tunneling frequency ${\nu }_{\pm }=({E}_{{1}_{01}^{-}}-{E}_{{1}_{01}^{+}})/h={\epsilon }_{\pm }/h$ (see Fig. 1). Due to the quantum tunneling, the created chiral wavepacket does not have a persistent chirality. However, the polarization induced by the M6WM experiment (Eq. (1)) is proportional to the total magnitude of the ∣*ee*∣ induced by the first three pulses. The probed *S*_M6WM_ with an amplitude of 13–14 μV indicates that a non-zero ∣*ee*∣ was created in the rotational state $\left|{1}_{01}\right\rangle$, and the M6WM approach allows us to measure the magnitude of the induced enantiomeric enrichment.

In conclusion, our experiment successfully steered a quantum racemic mixture of two interconvertible enantiomers off-equilibrium in the rotational state of interest by exploiting five microwave radiation pulses from three mutually orthogonal polarizations. This result demonstrates that transient enantiomeric excess can be induced in a quantum tunneling system with a suitable pulse sequence of resonant microwave fields. This provides an approach for manipulating molecular chirality in flexible molecules like BA.

## Methods

### Sample preparation

The commercial sample of benzyl alcohol (BA) was purchased from Sigma-Aldrich and used without further purification. Experiments were performed using a modified Fourier transform microwave (FTMW) spectrometer in Hamburg, as reported elsewhere^12^, and a sketch is provided in Fig. 2. The commercially available sample of BA was placed in an internal sample reservoir close to the solenoid nozzle valve (Parker General Valve, Series 9) and maintained at 70 °C. The sample vapor was seeded by a neon buffer gas with a stagnation pressure of ~3 bar and supersonically expanded into the vacuum chamber via the pulsed valve operating at 6 Hz. The adiabatic expansion efficiently cooled the gas-phase sample to a *T*_rot_ of ~1–2 K. For each gas pulse, the molecular ensemble was polarized with a series of eight pulse sequences, and 40 μs of FID were recorded following each excitation and averaged on an oscilloscope. After Fourier transformation, the typical frequency resolution of the spectrum is about 25 kHz, giving a full-width at half maximum (FWHM) linewidth of about 60 kHz.

### Optimal pulse conditions

To achieve the optimal condition of the proposed M6WM scheme for the population transfer, pulse #1 should be a *π*/2-pulse, while the other four pulses (#2 – #5) should be *π*-pulses. Therefore, nutation curves were performed for each excitation pulse to investigate the evolution of the signal amplitude over the pulse duration in a single-photon excitation experiment. Of them, the power of pulses #1 and #4, #2 and #5, and #3 was amplified to 40 W, 50 W and 3 W, respectively, using solid-state amplifiers (SSA), matching the setup in the subsequent M6WM experiment. The resulting nutation curves are shown in Supplementary Fig. 8, and the obtained optimal durations are summarized in Table 1.

### M6WM experiments

To achieve a state-specific enantiomer enrichment, the proposed M6WM experiment was carried out. Using the results of the nutation curves, the five pulses with optimal durations were generated with a two-channel arbitrary waveform generator (AWG), where pulses #1, #3, and #4 are from channel 1 (CH1), and pulses #2 and #5 are from channel 2 (CH2), as illustrated in Fig. 3a. As the polarization direction of pulse #3 needs to be orthogonal to pulses #1 and #4, a single pole double throw (SPDT) pin-diode switch was utilized to feed the signals into the dedicated SSAs. After amplification, the microwave pulses were broadcast into the vacuum chamber to interact with the molecules from three orthogonal polarization directions via two dual-polarization horn antennae. In order to explore the phase dependence of the signal induced from the M6WM scheme, the phase of each signal in the pulse sequence was systematically changed in steps of 18°. The free induction decays (FIDs) of the molecular response were recorded and averaged in the time domain and Fourier transformed into the frequency domain after applying a Gaussian window function. Each phase scan was repeated five times, in which 20,000 FIDs were averaged for each phase point. More details can be found in the Supplementary Information.

## Supplementary information

Supplementary Information

## Data Availability

All the data that support the findings of this study are available within the main text and the Supplementary Information file, and also available from the corresponding author on request.

## Code availability

The Python scripts used in this study are provided in the Supplementary Information.
