# Supplementary material for: Inducing transient enantiomeric excess in a molecular quantum racemic mixture with microwave fields
Source: Nat Commun. 2023 Feb 20;14:934. doi: 10.1038/s41467-023-36653-3 (PMC9941128; doi:10.1038/s41467-023-36653-3)
Supplement: Supplementary file 1 — Supplementary Information [file 41467_2023_36653_MOESM1_ESM.pdf]

Supplementary information:

Inducing transient enantiomeric excess in a molecular quantum racemic mixture with microwave fields

W. Sun,[†,‡] D. S. Tikhonov,[†,‡] H. Singh,[†,‡] A.L. Steber,[†,‡,¶] C. Pérez,[†,‡,¶] and M. Schnell*,[†,‡]

†*Deutsches Elektronen-Synchrotron DESY, Notkestr. 85, 22607 Hamburg, Germany*

‡*Institute of Physical Chemistry, Christian-Albrechts-Universität zu Kiel, Max-Eyth-Str. 1, 24118 Kiel, Germany*

¶*Current address: Departamento de Química Física y Química Inorgánica, Facultad de Ciencias-I.U. CINQUIMA, Universidad de Valladolid, E-47011 Valladolid, Spain.*

E-mail: melanie.schnell@desy.de

Contents

1 Supplementary theoretical details **4**

1.1 Phase dependence in the Rabi cycle . . . 4

1.2 Optimal pulse conditions . . . 5

1.3 Effect of the thermal population . . . 7

1.4 Physical interpretation of the cycle . . . 9

1.5 Proof of a closed cycle in the case of two large-amplitude motions . . . 10

2 Supplementary experimental details **14**

2.1 Experimental set-up . . . 14

2.2 Pulse durations at the Rabi flip angles of $\pi/2$ and π . . . 17

2.3 Microwave six-wave mixing (M6WM) experiments . . . 19

2.4 Testing of direct excitation by pulses from single amplifiers . . . 28

3 Supplementary data analysis **30**

3.1 Windowed Fourier Transformation . . . 30

3.1.1 Gaussian filter . . . 30

3.1.2 Rectangular filter . . . 31

3.1.3 Computational procedure . . . 31

3.1.4 Script for the analysis . . . 31

3.2 Description of the interference patterns . . . 37

3.2.1 General formulas . . . 37

3.2.2 The case of two interfering signals . . . 38

3.2.3 The case of more than two interfering signals . . . 40

3.3 Fitting of the interference patterns . . . 40

4 Supplementary experimental data **47**

4.1 Phase scan for pulse #1 (9178 MHz) . . . 47

4.2 Phase scan for pulse #2 (4373 MHz) . . . 49

4.3 Phase scan for pulse #3 (5293 MHz) . . . 52

4.4 Phase scan for pulse #4 (9231 MHz) . . . 54

4.5 Phase scan for pulse #5 (3459 MHz) . . . 57

1 Supplementary theoretical details

1.1 Phase dependence in the Rabi cycle

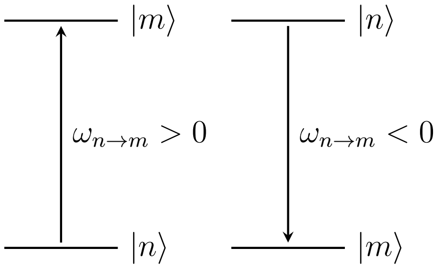

Supplementary Figure 1: Scheme for a two-level system with a coupled resonant field. The left case represents the absorption of the photon, while the right one is the stimulated emission.

For benzyl alcohol (BA), we will treat the transitions separately as a two-level state system coupled to the resonant electromagnetic field (Supplementary Figure 1). Such quantum system undergoes Rabi oscillations,[1,2] and we need to have the explicit phase dependence of the resulting wavefunction on the electric field phase in order to derive the phase dependence of the resulting polarization. For clarity, we will derive it from the beginning.[2]

Suppose we have two levels, $|m\rangle$ and $|n\rangle$ with energies E_m and E_n, respectively. The external field is oscillating with the resonant $|n\rangle \rightarrow |m\rangle$ transition angular frequency $\omega_{n\rightarrow m} = (E_m - E_n)/\hbar$. Let us assume that the electric field is $\mathcal{E}(t) = \mathbf{E}_0 \cos(\omega_{n\rightarrow m} t + \varphi)$, where φ is the carrier phase and $\mathbf{E}_0$ is the constant amplitude, carrying information of both maximal field strength and polarization. The time-dependent Schrödinger equation to solve is $i\hbar\frac{\partial}{\partial t}|\psi\rangle = \hat{H}|\psi\rangle$ with the Hamiltonian $\hat{H} = \hat{H}_0 + \hat{W}$. Here, $\hat{H}_0$ is the unperturbed Hamiltonian of the two-level system (i.e., $\hat{H}|m\rangle = E_m|m\rangle$ and $\hat{H}|n\rangle = E_m|n\rangle$), and $\hat{W} = \mathbf{E}(t) \cdot \hat{\boldsymbol{\mu}}$ describes the interaction of the system with the field in the dipole approximation. Taking the form of the wavefunction to be

$$|\psi(t)\rangle = \exp(-iE_m t/\hbar) c_m(t)|m\rangle + \exp(-iE_n t/\hbar) c_n(t)|n\rangle \ ,$$

and conditions of $\langle m|m\rangle = \langle n|n\rangle = 1$ and $\langle m|n\rangle = 0$, the Schrödinger equation will be converted to

$$\begin{cases} i\dot{c}_m = \frac{\mathbf{E}_0 \boldsymbol{\mu}_{mn}}{\hbar} \cos(\omega_{n\rightarrow m} t + \varphi) \cdot \exp(i\omega_{n\rightarrow m} t) c_n, \\ i\dot{c}_n = \frac{\mathbf{E}_0 \boldsymbol{\mu}_{mn}}{\hbar} \cos(\omega_{n\rightarrow m} t + \varphi) \cdot \exp(-i\omega_{n\rightarrow m} t) c_m, \end{cases}$$

where $\boldsymbol{\mu}_{mn} = \langle m|\hat{\boldsymbol{\mu}}|n\rangle = \boldsymbol{\mu}_{nm}$. Then we apply the rotating wave approximation (RWA) by taking the

cosine as $\cos(x) = (\exp(ix) + \exp(-ix))/2$ and ignoring the fast oscillating term. This will lead to a simplified system of equations:

$$\begin{cases} i\dot{c}_m = \frac{\Omega_{mn}}{2}\exp(-is_{n\to m}\varphi)c_n, \\ i\dot{c}_n = \frac{\Omega_{mn}}{2}\exp(+is_{n\to m}\varphi)c_m, \end{cases} \tag{1}$$

$\Omega_{mn} = \frac{\boldsymbol{\mu}_{mn}\mathbf{E}_0}{\hbar}$ is the Rabi frequency and $s_{n\to m} = \mathrm{sign}(\omega_{n\to m})$, meaning $s_{n\to m} = +1$ if $\omega_{n\to m} > 0$ and $s_{n\to m} = -1$ if $\omega_{n\to m} < 0$. This electromagnetic field phase sign ambiguity appears from the choice of the resonant cosine term in the RWA.

The solutions of the Equations 1 with initial conditions of $|c_n(0)|^2 = 1$ and $c_m(0) = 0$ are

$$\begin{cases} c_n(t) = \exp(i\phi_n^{(0)}) \cdot \cos\left(\frac{\Omega_{mn}t}{2}\right), \\ c_m(t) = -i\exp(i\phi_n^{(0)} - is_{n\to m}\varphi) \cdot \sin\left(\frac{\Omega_{mn}t}{2}\right), \end{cases}$$

where $\phi_n^{(0)}$ is the initial phase (at $t = 0$) of the state $|n\rangle$. The final state after interaction for a time $t = \tau$ with the external resonant field is

$$\psi(t) = \exp(i\phi_n^{(0)}) \cdot \left[\exp\left(-\frac{iE_nt}{\hbar}\right) \cdot \cos\left(\frac{\Omega_{mn}\tau}{2}\right)|n\rangle - i\exp\left(-is_{n\to m}\varphi - \frac{iE_mt}{\hbar}\right) \cdot \sin\left(\frac{\Omega_{mn}\tau}{2}\right)|m\rangle\right] \tag{2}$$

By applying this wavefunction form to the sequence of the pulses in the microwave six-wave mixing (M6WM) scheme (Supplementary Figure 2), one can follow the dynamics of the system starting from the initial state, with the rule

- when going up in energy ($\omega_{n\to m} > 0$), $s_{n\to m} = +1$,
- when going down in energy ($\omega_{n\to m} < 0$), $s_{n\to m} = -1$.

1.2 Optimal pulse conditions

The general form of the sequence used in the experiment is given in Supplementary Figure 2. It consists of five subsequent pulses, which transfer population between states labeled $|n_\pm\rangle$ for generality reasons:

1. $|0_-\rangle \to |1_-\rangle$ (c-type intrastate transition with Rabi frequency Ω_1 and duration τ_1, $\omega_1 = \omega_{0_-\to 1_-}$, phase of the pulse is φ_1, $s_1 = +1$),
2. $|1_-\rangle \to |2_+\rangle$ (b-type interstate transition with Rabi frequency Ω_2 and duration τ_2, $\omega_2 = \omega_{1_-\to 2_+}$, phase of the pulse is φ_2, $s_2 = -1$),

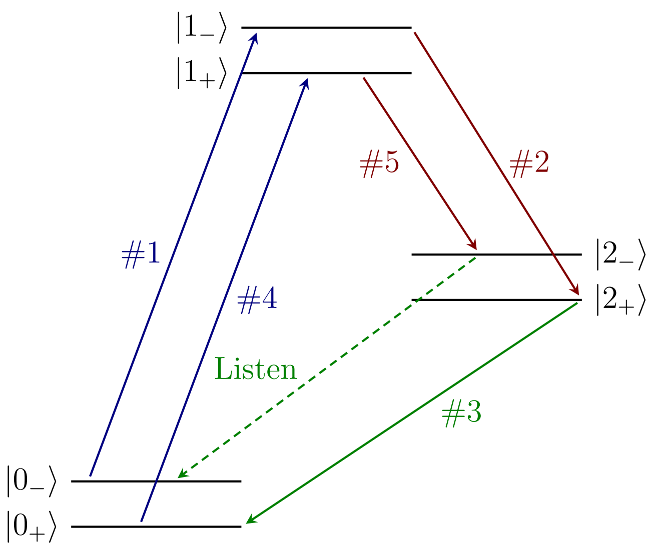

Supplementary Figure 2: General scheme of the pulse sequence. The horizontal lines denote energy levels, the y-axis is the energy, arrows are the transitions, and the numbers of the arrows are the numbers of the pulses in the sequence.

3. $|2_+\rangle \to |0_+\rangle$ (a-type intrastate transition with Rabi frequency Ω_3 and duration τ_3, $\omega_3 = \omega_{2_+ \to 0_+}$, phase of the pulse is φ_3, $s_3 = -1$),

4. $|0_+\rangle \to |1_+\rangle$ (c-type intrastate transition with Rabi frequency Ω_4 and duration τ_4, $\omega_4 = \omega_{0_+ \to 1_+}$, phase of the pulse is φ_4, $s_4 = +1$),

5. $|1_+\rangle \to |2_-\rangle$ (a-type interstate transition with Rabi frequency Ω_5 and duration τ_5, $\omega_5 = \omega_{1_+ \to 2_-}$, phase of the pulse is φ_5, $s_5 = -1$).

The types of transitions we used here match the experimental scheme, which will be described in Supplementary Section 2, but in general they can be switched to correspond to any closed cycle of six transitions. This pulse sequence induces a macroscopic polarization for $|2_-\rangle \to |0_-\rangle$ (listen) transition with frequency $\omega = \omega_{0_- \to 2_-}$.

Sequential evaluation of the wavefunction using Equation 2 leads to the final state

$$|\psi\rangle = \exp\left(i\phi_{0_-}^{(0)} - \frac{iE_{0\_}t}{\hbar}\right) \cdot \left[\overbrace{\cos\left(\frac{\Omega_1\tau_1}{2}\right)}^{c_{0_-}} |0_-\rangle \underbrace{-i\exp(-i\omega t + i\Phi)\prod_{k=1}^{5}\exp(-is_k\varphi_k)\sin\left(\frac{\Omega_k\tau_k}{2}\right)|2_-\rangle}_{c_{2-}} + \dots \right],$$

where Φ is the total phase accumulated during the cycle.

The observable in the experiment is the macroscopic polarization for the listen transition ($|2_-\rangle \to |0_-\rangle$)

described by the operator

$$\hat{\mathbf{P}}_{0_-2_-} = \boldsymbol{\mu}_{0_-2_-} \cdot (|0_-\rangle\langle 2_-| + |2_-\rangle\langle 0_-|) \ .$$

Therefore, the time evolution of this polarization is

$$\langle \mathbf{P}_{0_-2_-} \rangle = \langle \psi | \hat{\mathbf{P}}_{0_-2_-} | \psi \rangle = 2\boldsymbol{\mu}_{0_-2_-} \mathrm{Re}(c^*_{0_-} c_{2_-}) =$$
$$= -\boldsymbol{\mu}_{0_-2_-} \sin(\Omega_1 \tau_1) \cdot \left(\prod_{k=2}^{5} \sin\left(\frac{\Omega_k \tau_k}{2} \right) \right) \cdot \sin(\omega t + \sum_{k=1}^{5} s_k \varphi_k - \Phi) \quad (3)$$

From this equation, we can deduce the optimal set of conditions to maximize the listen transition intensity.

- To ensure the proper match to the types of rotational transitions involved in the cycle, the polarizations of the pulses should be as follows:
  - pulses #1 and #4 should be in the same direction (say, x),
  - pulses #2 and #5 should be in the same direction, and orthogonal to #1 and #4 (say, y),
  - pulse #3 and the listen transition should have the polarization orthogonal to the aforementioned (here, z).
- The duration of the pulses should fulfill the following conditions:
  - the first pulse (#1) should be a $\pi/2$-pulse ($\Omega_1\tau_1 = \pi/2$), to maximize $\sin(\Omega_1\tau_1)$.
  - the other pulses (#2–#5) should be π-pulses ($\Omega_k\tau_k = \pi$, $k = 2, 3, 4, 5$), to maximize the $\sin(\Omega_k\tau_k/2)$ terms.

  The interpretation of this condition is the following: the first pulse splits the population in state $|0_-\rangle$ into two equal parts, one left in $|0_-\rangle$ till the end of the experiment, and the second is being transferred from state $|1_-\rangle$ to $|2_-\rangle$ using the pulses #2 – #5.

1.3 Effect of the thermal population

Although the signal above was derived for the system starting from the state $|0_-\rangle$, there will also be molecules populated in the other rotational states, particularly the state $|1_-\rangle$, in a real supersonically expanded molecular ensemble. Therefore, whenever the cycle given in Supplementary Figure 2 will be driven by the pulses, at the same time another cycle will be driven, starting from state $|1_-\rangle$ (Supplementary Figure 3). Other possible cycles can be neglected because the pulse sequence will not fulfill the connectivity requirements for the states and the resonance conditions.

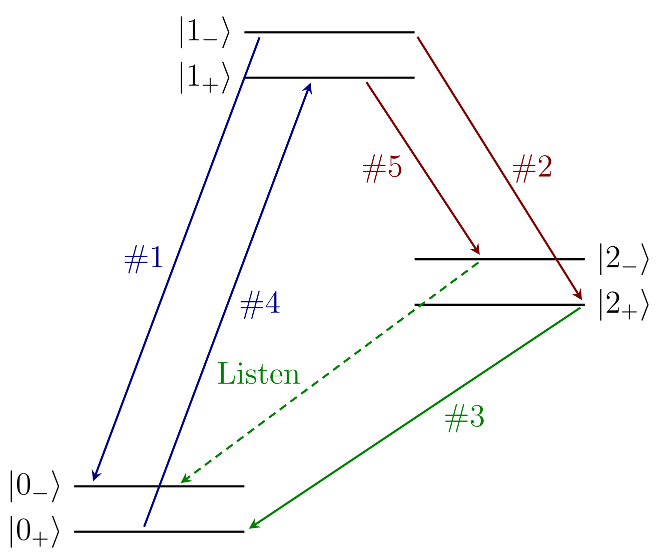

Supplementary Figure 3: A cycle driven from the excited state. The notations are the same as in Supplementary Figure 2.

The difference between the two possible cycles (Supplementary Figures 2 and 3) lies in the effect of the first pulse: while in the first one the population from state $|0_-\rangle$ is being transferred to $|1_-\rangle$, in the second the population from $|1_-\rangle$ is being transferred to $|0_-\rangle$. The final state of the second cycle will be (similar to the previous section):

$$|\psi'\rangle = \exp\left(i\phi_{0_-}^{(0)} - \frac{iE_{0\_}t}{\hbar}\right)\cdot\Big[\overbrace{-i\sin\left(\frac{\Omega_1\tau_1}{2}\right)\cdot\exp(+is_1\varphi_1)}^{c'_{0_-}}|0_-\rangle + \\ + \underbrace{\exp(-i\omega t + i\Phi)\cos\left(\frac{\Omega_1\tau_1}{2}\right)\prod_{k=2}^{5}\exp(-is_k\varphi_k)\sin\left(\frac{\Omega_k\tau_k}{2}\right)}_{c'_{2-}}|2_-\rangle + \dots\Big].$$

The sign before s_1 in c'_{0_-} coefficient is inverted because, unlike in the initial scheme (Supplementary Figure 2), the system is being transferred from a state higher in energy to the state lower in energy. The resulting signal (similar to Equation 3) will thus be

$$\langle\psi'|\hat{\mathbf{P}}_{0\_2\_}|\psi'\rangle = 2\boldsymbol{\mu}_{0\_2\_}\mathrm{Re}((c'_{0_-})^* c'_{2_-}) = \\ = +\boldsymbol{\mu}_{0\_2\_}\sin(\Omega_1\tau_1)\cdot\left(\prod_{k=2}^{5}\sin\left(\frac{\Omega_k\tau_k}{2}\right)\right)\cdot\sin(\omega t + \sum_{k=1}^{5}s_k\varphi_k - \Phi) = -\langle\psi|\hat{\mathbf{P}}_{0\_2\_}|\psi\rangle \quad (4)$$

In other words, the two cycles, that start from the lower state $|0_-\rangle$ (Supplementary Figure 2) and from the upper state $|1_-\rangle$ (Supplementary Figure 3), respectively, are giving rise to coherent signals that are

opposite in the phase. Therefore, the temperature-averaged signal can be written as

$$\langle\langle \mathbf{P}_{0_-2_-}\rangle\rangle_T = n_{0_-} \cdot \langle\psi|\hat{\mathbf{P}}_{0_-2_-}|\psi\rangle + n_{1_-} \cdot \langle\psi'|\hat{\mathbf{P}}_{0_-2_-}|\psi'\rangle = (n_{0_-} - n_{1_-}) \cdot \langle\psi|\hat{\mathbf{P}}_{0_-2_-}|\psi\rangle \ ,$$

where n_x is the population in the state $|x\rangle$. Therefore, to observe the M6WM signal, the molecular ensemble must have a nonzero population difference ($|n_{0_-} - n_{1_-}| > 0$) between the two states that are being influenced by the first pulse of the sequence, similar to non-chiral coherence Fourier transform microwave (FTMW) spectroscopy.

1.4 Physical interpretation of the cycle

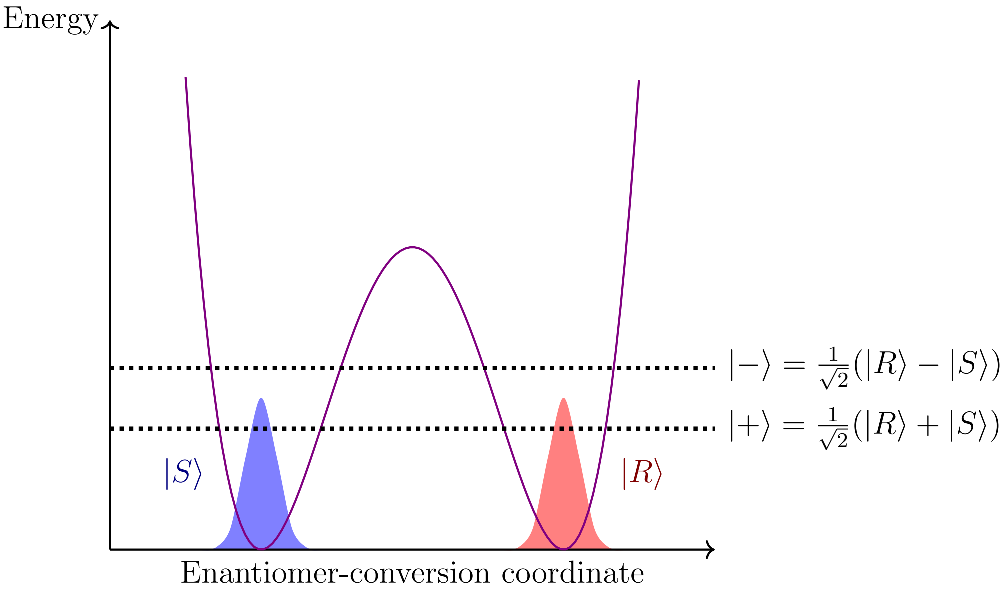

Supplementary Figure 4: Scheme representing the double-well potential of the transiently chiral molecule.

Transiently chiral molecules, such as BA, can be described by a double-well potential (Supplementary Figure 4): two enantiomers, R and S, correspond to the two minima of the potential with a pathway connecting them through an achiral transition state.[3–6] In the case of BA, the enantiomer-conversion motion corresponds to the internal rotation of the $-CH_2OH$ group with respect to the phenyl ring. The two enantiomers would correspond to a vibrational wavepacket isolated in a single well, namely $|S\rangle$ and $|R\rangle$. However, if tunneling occurs on the experimental timescale, the degeneracy of the single-well localized states is lifted, resulting in symmetric ($|+\rangle$) and antisymmetric ($|-\rangle$) eigenfunctions of the Hamiltonian:

$$|\pm\rangle = \frac{1}{\sqrt{2}}(|R\rangle \pm |S\rangle) \ .$$

If the inversion motion is feasible, i.e., tunneling occurs within the timescale of the experiment, the molecules will generally exist in the $|\pm\rangle$ states, so to make the molecule chiral, one has to create a 50/50 superposition of the $|+\rangle$ and $|-\rangle$ states, such as the entangled states

$$\begin{cases} |R\rangle = \frac{1}{\sqrt{2}}(|+\rangle + |-\rangle) \ , \\ |S\rangle = \frac{1}{\sqrt{2}}(|+\rangle - |-\rangle) \ . \end{cases}$$

The first three pulses of the sequence given in Supplementary Figure 2 create this superposition. This result can be illustrated via the usage of the enantiomeric excess (ee) observable. By definition, the ee is the difference between the amounts of the R and S enantiomers.[7] In quantum mechanics, this observable is given by the operator[3–5]

$$\hat{ee} = |R\rangle\langle R| - |S\rangle\langle S| = |+\rangle\langle -| + |-\rangle\langle +| \ ,$$

i.e., by the coherence between the $|\pm\rangle$ states. The wavefunction after pulses #1, #2, and #3 is given by

$$|\psi_3\rangle = \exp\left(i\phi_{0_-}^{(0)} - \frac{iE_{0_-}t}{\hbar}\right) \cdot \left[\overbrace{\cos\left(\frac{\Omega_1\tau_1}{2}\right)}^{c_{0_-}} |0_-\rangle \underbrace{+i\exp(+i\omega_{\pm}t + i\Phi') \prod_{k=1}^{3} \exp(-is_k\varphi_k) \sin\left(\frac{\Omega_k\tau_k}{2}\right) |0_+\rangle}_{c_{0+}} + \ldots \right] \ ,$$

where $\omega_{0_+\to 0_-} = (E_{0_-} - E_{0_+})/\hbar$. Therefore, the ee for the rotational state $|0\rangle$ is

$$\langle ee\rangle = \langle\psi_3|\hat{ee}|\psi_3\rangle = \overbrace{-\sin(\Omega_1\tau_1)\left(\prod_{k=2}^{3}\sin\left(\frac{\Omega_k\tau_k}{2}\right)\right)}^{\propto|ee|} \cdot \sin(\omega_{0_+\to 0_-}t - \sum_{k=1}^{3} s_k\varphi_k + \Phi')$$

This is a solution that oscillates at the tunneling frequency $\omega_{0_+\to 0_-}$ with the amplitude $|ee|$. While staying in this superposition, the system continuously interconverts between the two enantiomers. However, the subsequent two pulses (#4 and #5), creating the coherent signal of the listen transition, allow to probe the magnitude of the ee created, as the Equation 3 can be rewritten as

$$\langle \mathbf{P}_{0_-2_-}\rangle = -\boldsymbol{\mu}_{0_-2_-} \cdot |ee| \cdot \left(\prod_{k=4}^{5}\sin\left(\frac{\Omega_k\tau_k}{2}\right)\right) \cdot \sin(\omega t + \sum_{k=1}^{5} s_k\varphi_k - \Phi) \ .$$

1.5 Proof of a closed cycle in the case of two large-amplitude motions

With respect to the potential energy surface, BA exists in the form of four equivalent structures as depicted in Supplementary Figure 5. Let us denote them as $|R_u\rangle$, $|S_u\rangle$, $|R_d\rangle$, and $|S_d\rangle$.[19] Here, R/S denote the

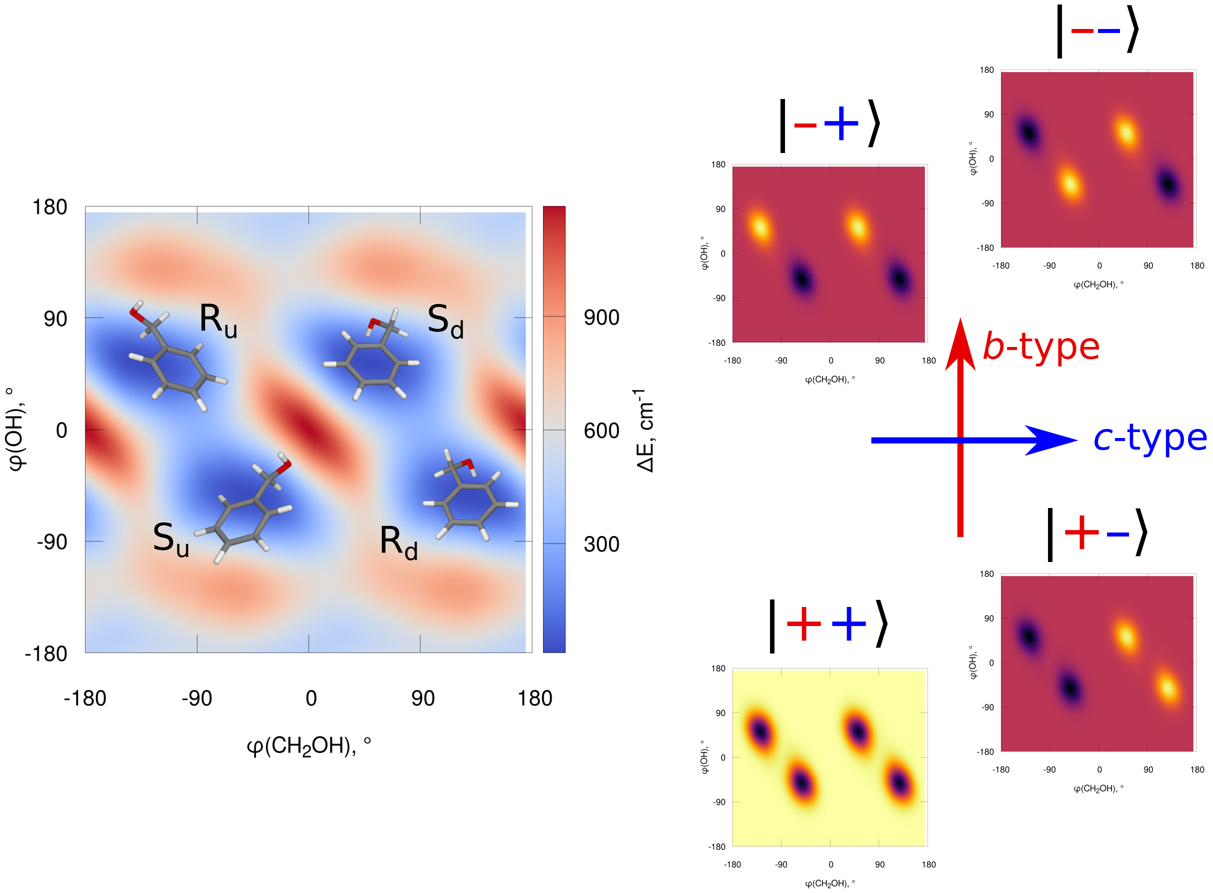

Supplementary Figure 5: Potential energy landscape for internal rotation in the BA molecule and the ground vibrational state wavefunctions appearing from the tunneling splitting. The potential energy surface was computed at the B3LYP-D3BJ/def2-TZVPP level of theory[8–12] using Orca 4.[13] The wavefunctions were computed the same way as in the Refs. Bataev et al.[14], Godunov et al.[15], Bokarev et al.[16], Kudich et al.[17]. The predicted splitting for the *b*-type transition was 2.5 GHz (experimental value is 0.46 GHz[18]), while for the *c*-type, the predicted splitting was 4 kHz.

stereochemical configuration of BA, whilst u/d denote "up" and "down", referring to the relative direction of the $-CH_2OH$ group with respect to the phenyl ring. These four structures can interconvert through two large amplitude motions (LAMs).

- The first motion, that is also experimentally observed in BA using rotational spectroscopy,[19,20] corresponds to the motion of $-CH_2OH$ staying on the same side of the phenyl ring (i.e., $|R_{u/d}\rangle \leftrightarrow |S_{u/d}\rangle$). This LAM has a relatively low experimental barrier of around 280 cm^{-1} and causes a change of the dipole moment along the *b*-axis of the molecule. Therefore, interstate transitions associated with this motion are observed for *b*-type transitions.

- The second motion has a significantly higher barrier predicted theoretically to be around 700 cm^{-1}. This motion of the $-CH_2OH$ takes place between opposite sides of the phenyl ring (i.e., $|R_{u/d} \rightarrow |S_{d/u}\rangle$), and thus changes the sign of the *c*-component of the dipole moment of the molecule. Therefore, interstate transitions associated with this motion are observed for *c*-type transitions. In

BA, this type of splitting was not observed in the experiment on the high-resolution cavity FTMW spectrometer,[19] however, for its derivative (3,5-difluorobenzyl alcohol), it was seen.[20]

Due to these two LAMs, the ground vibrational state of BA is split into four vibrational sublevels: $| \pm \pm\rangle$, where the first label denotes the parity of the b-type motion and the second of the c-type motion (see Supplementary Figure 5).

- The lowest energy state is

$$|++\rangle = \frac{1}{2}(|R_u\rangle + |S_u\rangle + |R_d\rangle + |S_d\rangle) \ .$$

- The second state in energy is

$$|+-\rangle = \frac{1}{2}(|R_u\rangle + |S_u\rangle - |R_d\rangle - |S_d\rangle) \ .$$

  This is because the splitting caused by the c-type motion is several orders of magnitude smaller than for the b-type motion.

- The third state in energy is

$$|-+\rangle = \frac{1}{2}(|R_u\rangle - |S_u\rangle - |R_d\rangle + |S_d\rangle) \ .$$

- And the highest energy ground vibrational state is

$$|--\rangle = \frac{1}{2}(|R_u\rangle - |S_u\rangle + |R_d\rangle - |S_d\rangle) \ .$$

Since there is no observable frequency difference between $| \pm +\rangle \leftrightarrow | \pm -\rangle$ c-type transitions, in our M6WM experiments, we will be driving two cycles simultaneously. The first one starts from the $|1_{01}^{-+}\rangle$ state, while the second cycle begins from $|1_{01}^{--}\rangle$ ($\pm\pm/\pm\mp$ again denote the parity of the wavefunction with respect to the first and the second motion).

$$\begin{array}{ccc}
2_{11}^{--} & \xrightarrow{(b)} & 2_{02}^{+-} \\
(c)\uparrow & & \downarrow(a) \\
1_{01}^{-+} & & 1_{01}^{+-} \\
(a)\uparrow & & \downarrow(c) \\
2_{02}^{-+} & \xleftarrow[(b)]{} & 2_{11}^{++}
\end{array}
\qquad
\begin{array}{ccc}
2_{11}^{-+} & \xrightarrow{(b)} & 2_{02}^{++} \\
(c)\uparrow & & \downarrow(a) \\
1_{01}^{--} & & 1_{01}^{++} \\
(a)\uparrow & & \downarrow(c) \\
2_{02}^{--} & \xleftarrow[(b)]{} & 2_{11}^{+-}
\end{array}$$

The first cycle would correspond to the formation of the following enantiopure entangled rovibrational states:

$$\begin{cases} |-+\rangle + |+-\rangle \propto |R_u\rangle - |R_d\rangle \ , \\ |-+\rangle - |+-\rangle \propto |S_d\rangle - |S_u\rangle \ . \end{cases}$$

The second cycle will give in turn:

$$\begin{cases} |++\rangle + |--\rangle \propto |R_u\rangle + |R_d\rangle \ , \\ |++\rangle - |--\rangle \propto |S_d\rangle + |S_u\rangle \ . \end{cases}$$

Therefore, despite of having two LAMs instead of one, we still will get closed cycles of levels driven by the M6WM pulse sequence. These independent cycles will also both produce the state of chosen induced chirality, similar to the case of the one LAM.

2 Supplementary experimental details

2.1 Experimental set-up

The experiments were carried out using the modified Fourier transform (CP-FTMW) spectrometer COMPACT.[21] The main schematic of the apparatus has been reported elsewhere,[22] and thus only a brief overview of the set-up, recent modifications, and the experimental procedures is given here. The commercially available sample of benzyl alcohol (BA) was placed in an internal sample reservoir close to the solenoid valve (Parker General Valve, Series 9) and was heated and maintained at 70 °C. The sample vapor was seeded in a neon buffer gas with a stagnation pressure of ∼3 bar and supersonically expanded into the vacuum chamber via the pulsed valve at a repetition rate of 6 Hz. In order to achieve a state-specific enantiomeric enrichment at the low temperature in the jet expansion, a five-pulse sequence was generated by a two-channel arbitrary waveform generator (AWG) and broadcast in the designated orthogonal polarizations via two dual-polarization horn antennae, as shown in Supplementary Figure 6. Following the theoretical considerations, the selected rotational energy levels and the pulse sequence are presented in Supplementary Figure 7. Pulses #1 and #4, #2 and #5, and #3 were amplified with 40 W, 50 W, and 3 W solid-state amplifiers, respectively. After the molecular ensamble was polarized by the excitation pulses, free induction decays (FIDs) of the electric field component of the emission signal were recorded and averaged in the time domain using a fast oscilloscope and Fourier transformed into the frequency domain employing a Gaussian window function, which will be described in Supplementary Section 3.1. To reduce the total measurement time, eight consecutive pulse sequences were performed on each supersonic gas expansion, making the effective repetition rate of the FID accumulations to be 48 Hz.

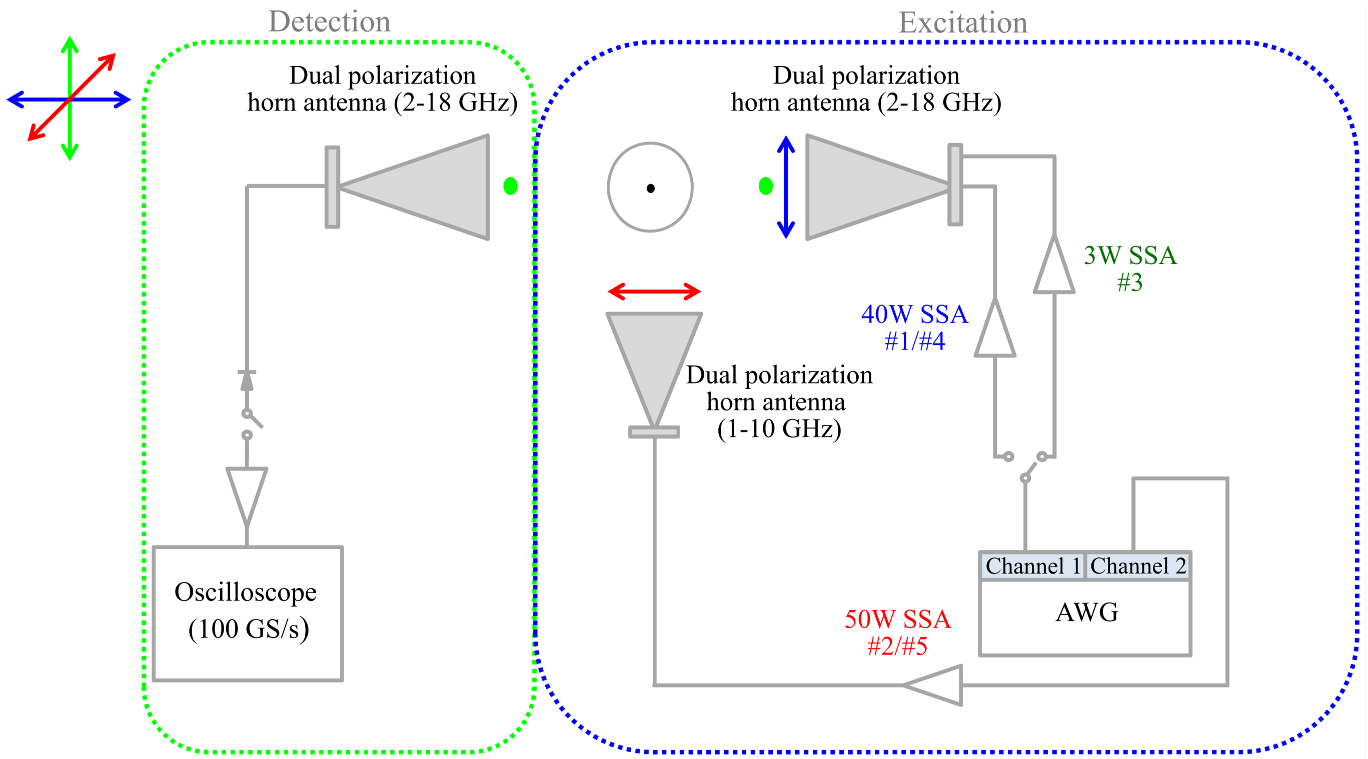

Supplementary Figure 6: The experimental set-up for the microwave six-wave mixing (M6WM) experiment.

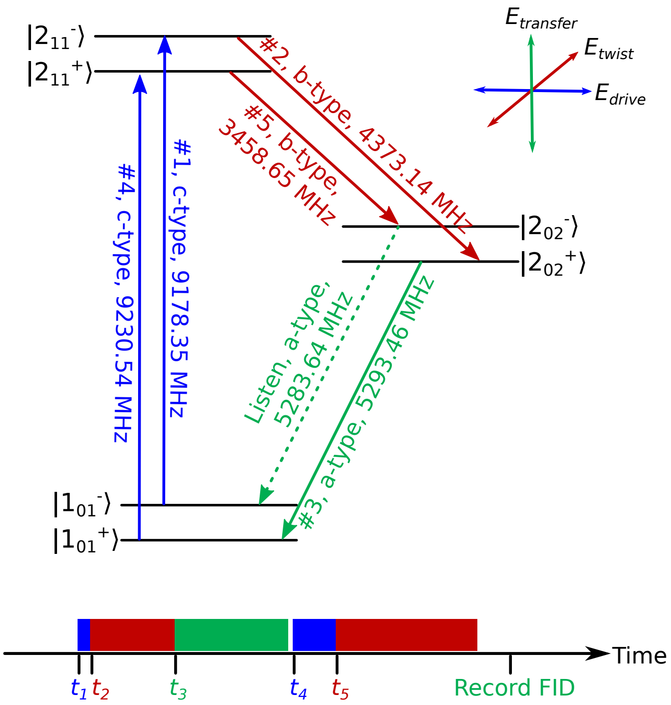

Supplementary Figure 7: The relevant rotational energy levels of benzyl alcohol (BA) and the corresponding M6WM pulse scheme to achieve chiral control of it. Each rotational state is denoted with $|J_{K_aK_c}{}^{\pm}\rangle$. The superposition of the two tunneling states $|+\rangle$ and $|-\rangle$ of the rotational state $|1_{01}\rangle$ is created by the first three entanglement pulses, which induces a Glauber state, which concentrates an oscillating wave package close to the classical trajectory (here oscillation between R and S).[23] Subsequently, in close analogy to a M3WM experiment, the last two pulses in the sequence induce a chiral signal at the frequency of the listen transition (5283.64 MHz), allowing the detection of the created enantiomeric excess in the $|1_{01}{}^{-}\rangle$ state.

2.2 Pulse durations at the Rabi flip angles of $\pi/2$ and π

The Rabi flip angle is defined as

$$\Omega_{\text{Rabi}} = \frac{\mu \cdot E}{\hbar} \cdot \tau \tag{5}$$

where μ denotes the transition dipole moment for the rotational transition, E and τ are the electric field amplitude and duration of the coherent microwave pulse. At the Rabi flip angle of $\pi/2$, a maximum coherence is induced at population equilibration, thereby maximizing the transition intensity, while at the angle of π, population inversion at complete coherence transfer within a 2-level system is achieved. Note that the effect of the M_J states is not treated here. We are currently implementing a setup based on circularly polarized microwave fields that will allow us to treat two thirds of the total M_J degeneracy.[24] As demonstrated in Supplementary Section 1.2, to achieve the optimal conditions for the population transfer, the first pulse (#1) in the proposed M6WM cycle should be a $\pi/2$-pulse, which creates the maximum coherence for the $|2_{11}{}^{-}\rangle \rightarrow |1_{01}{}^{-}\rangle$ transition, while pulses #2–#5 should be π-pulses. The population in the $|1_{01}{}^{-}\rangle$ state is thus split into two halves. One half stays till the end of the cycle, and the other half is transferred to the final $|2_{02}{}^{-}\rangle$ state through all other pulses (#2–#5) in the sequence.

Experimentally, the optimal pulse durations were determined via nutation curves for all five transitions by individually varying the durations of the single-frequency excitation pulses. The results are presented in Supplementary Figure 8. The pulse duration that induces the maximum signal intensity in each time scan corresponds to the $\pi/2$-pulse condition, and the duration for the π-pulse condition is taken as twice of the $\pi/2$-pulse length. The optimized durations from these single-photon excitation experiments are summarized in Supplementary Table 1 and were used in the M6WM experiments. Note that these are only effective values due to the manifold of M_J substates. This also explains the absence of clear minima indicating π conditions. Therefore, twice of the $\pi/2$ conditions were chosen as π conditions.

Supplementary Table 1: Optimized pulse durations for the excitation pulses for the M6WM experiments, obtained by individual measurements.

Pulse #	Frequency (MHz)	Type	Duration (μs)	Condition	Amplification power (W)
1	9178.35	c-type	0.2	$\pi/2$	40
2	4373.14	b-type	1.2	π	50
3	5293.46	a-type	1.6	π	3
4	9230.54	c-type	0.6	π	40
5	3458.65	b-type	2.0	π	50

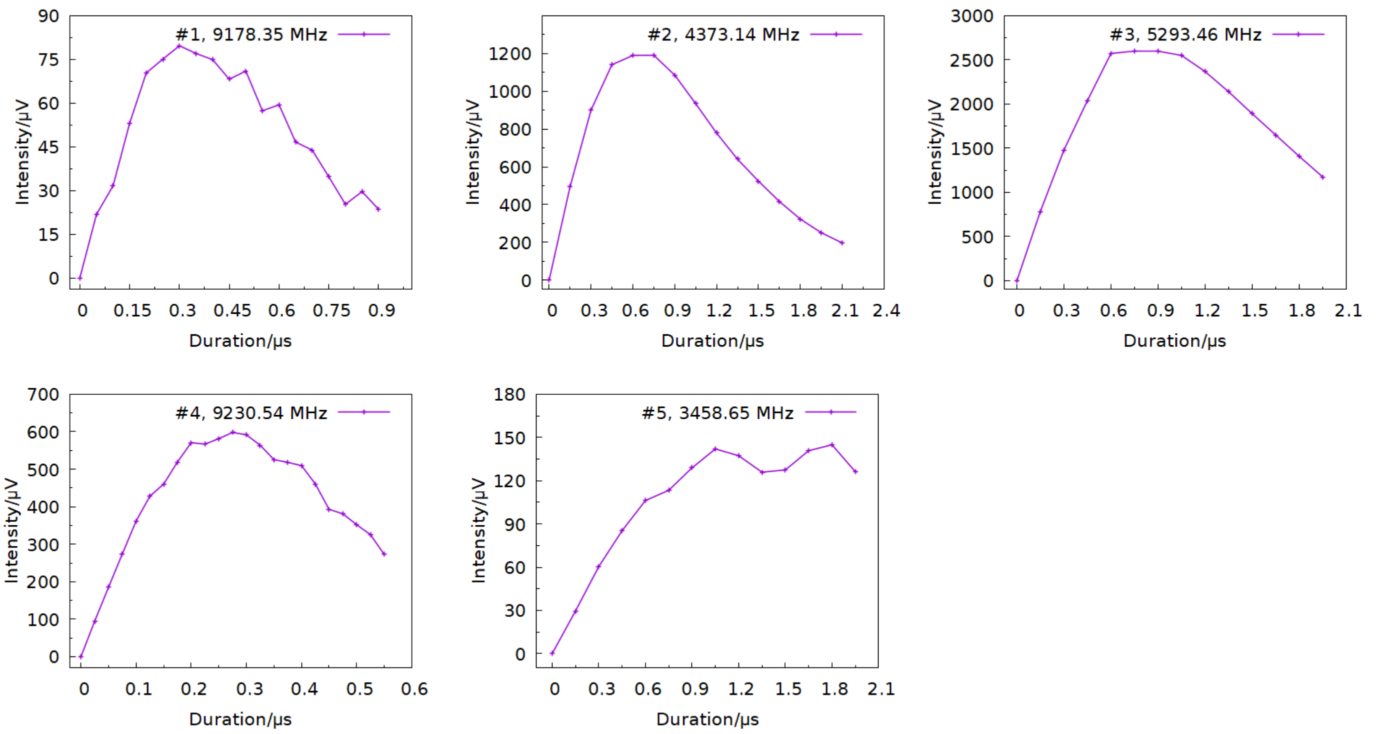

Supplementary Figure 8: Nutation curves for the five rotational transitions in the M6WM cycle. Each point on the curve is averaged from 20,000 FIDs, where the direct excitation and detection have the same polarization. Pulses #1 and #4 were amplified with the 40 W SSA, and the steps in the time scans are 0.05 and 0.025 μs correspondingly. Pulses #2 and #5 were amplified with the 50 W SSA, and the scan increments are 0.15 μs for both. Pulse #3 was amplified with the 3W SSA with scan increments of 0.15 μs.

2.3 Microwave six-wave mixing (M6WM) experiments

Following the nutation curve scans, the five pulses with the optimized durations were programmed into the two AWG channels, as depicted in Supplementary Figure 9. In the experiment, the relative phase of each excitation pulse was varied from 0° to 360° in steps of 18°, and the molecular response of the listen transition (5283.64 MHz) at every phase was recorded and averaged with 20,000 FIDs. Each set of the experiment was performed four times continuously on the same day to ensure phase stability and reproducibility. The results are presented in Supplementary Figures 10 to 14. The relative phase information corresponding to the scanned transition and the resulting listen transition were extracted from the time domain, as shown in Supplementary Figures 15 and 16. Note that, limited by the sampling rate of the AWG (12 GSa/s each channel), a frequency doubler was applied before the amplifier for pulses #1 and #4. Therefore, the programmed frequencies of these two pulses are only half of the frequencies of the transitions. Likewise, the programmed phase increments need to be halved before frequency doubling. After all five phase scans were completed, each of them was repeated once more to check the reproducibility. The comparison of these last repeats with the previous four measurements is shown in Supplementary Figure 17.

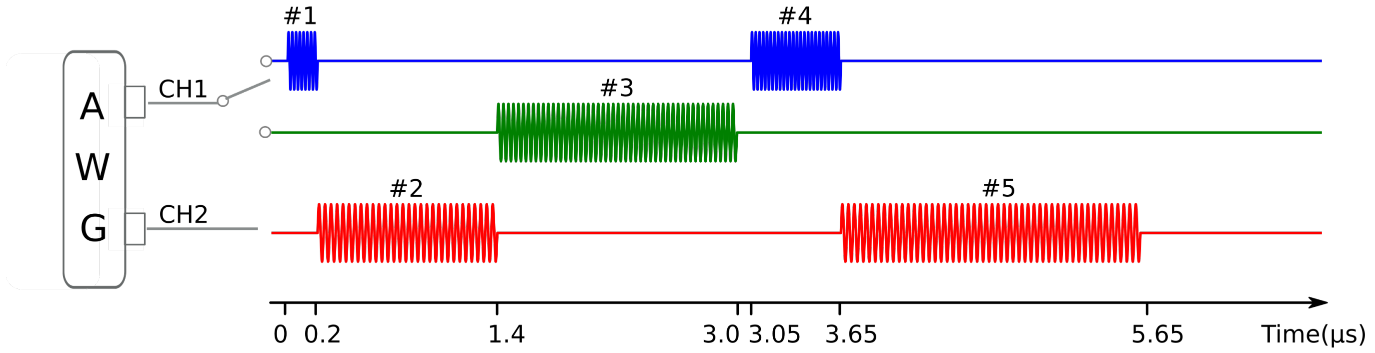

Supplementary Figure 9: Optimized pulse sequence for the M6WM experiment. Pulses #1, #3, and #4 are generated from channel 1 (CH1) of the AWG, pulses #2 and #5 are from channel 2 (CH2). As the polarization plane of pulse #3 should be orthogonal to #1 and #4, these signals are fed through the designated output of a single pole double throw (SPDT) pin diode switch. A delay of 0.05 μs between pulses #3 and #4 is applied to account for the switching time.

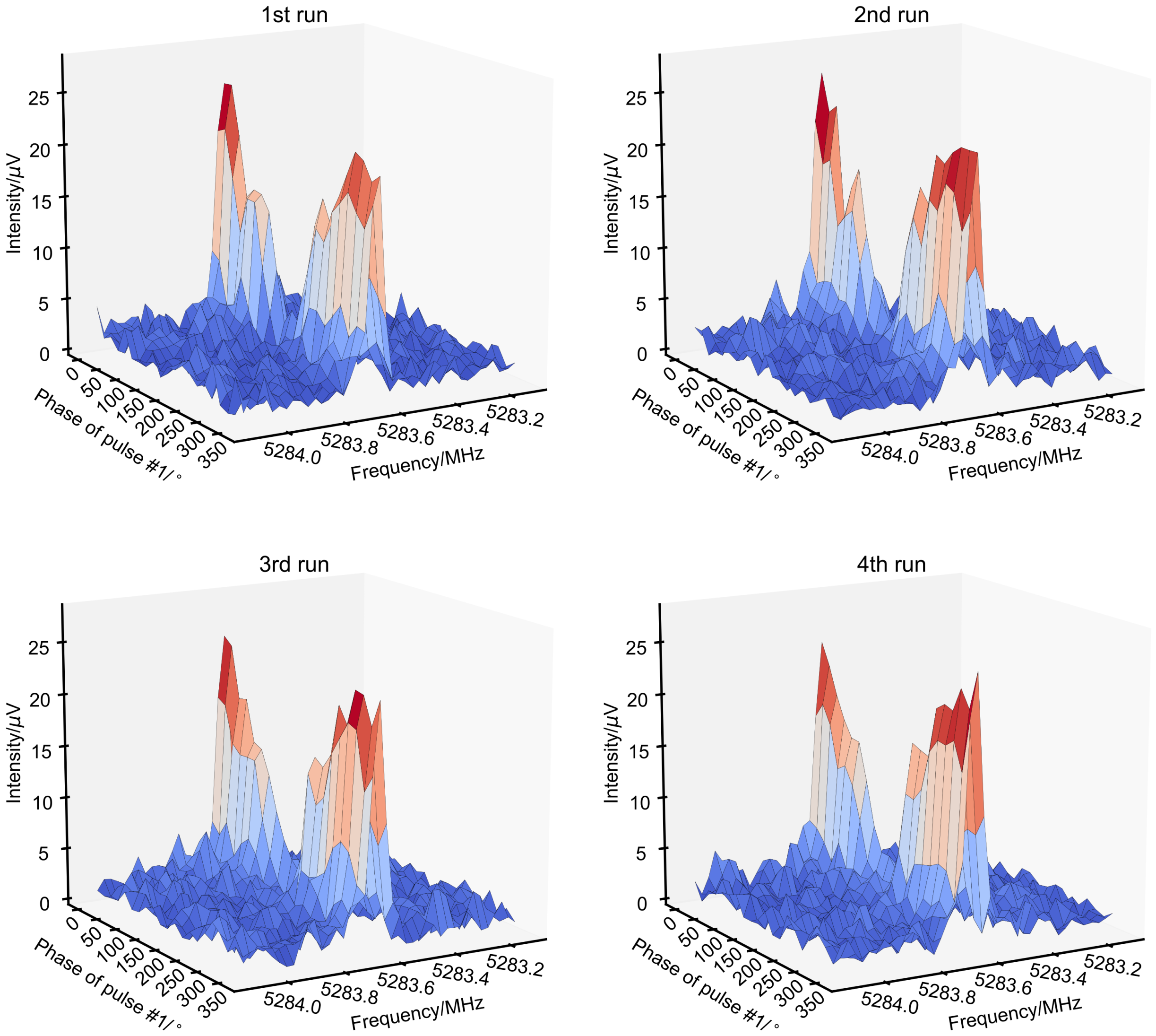

Supplementary Figure 10: 3D plot of the signal intensity of the listen transition at 5283.64 MHz while varying the phase of pulse #1, which corresponds to the $|2_{11}{}^{-}\rangle \rightarrow |1_{01}{}^{-}\rangle$ transition at 9178.35 MHz, in steps of 18°. The same experiment was performed four times continuously on the same day to ensure the reproducibility. Each step was averaged with 20,000 FIDs.

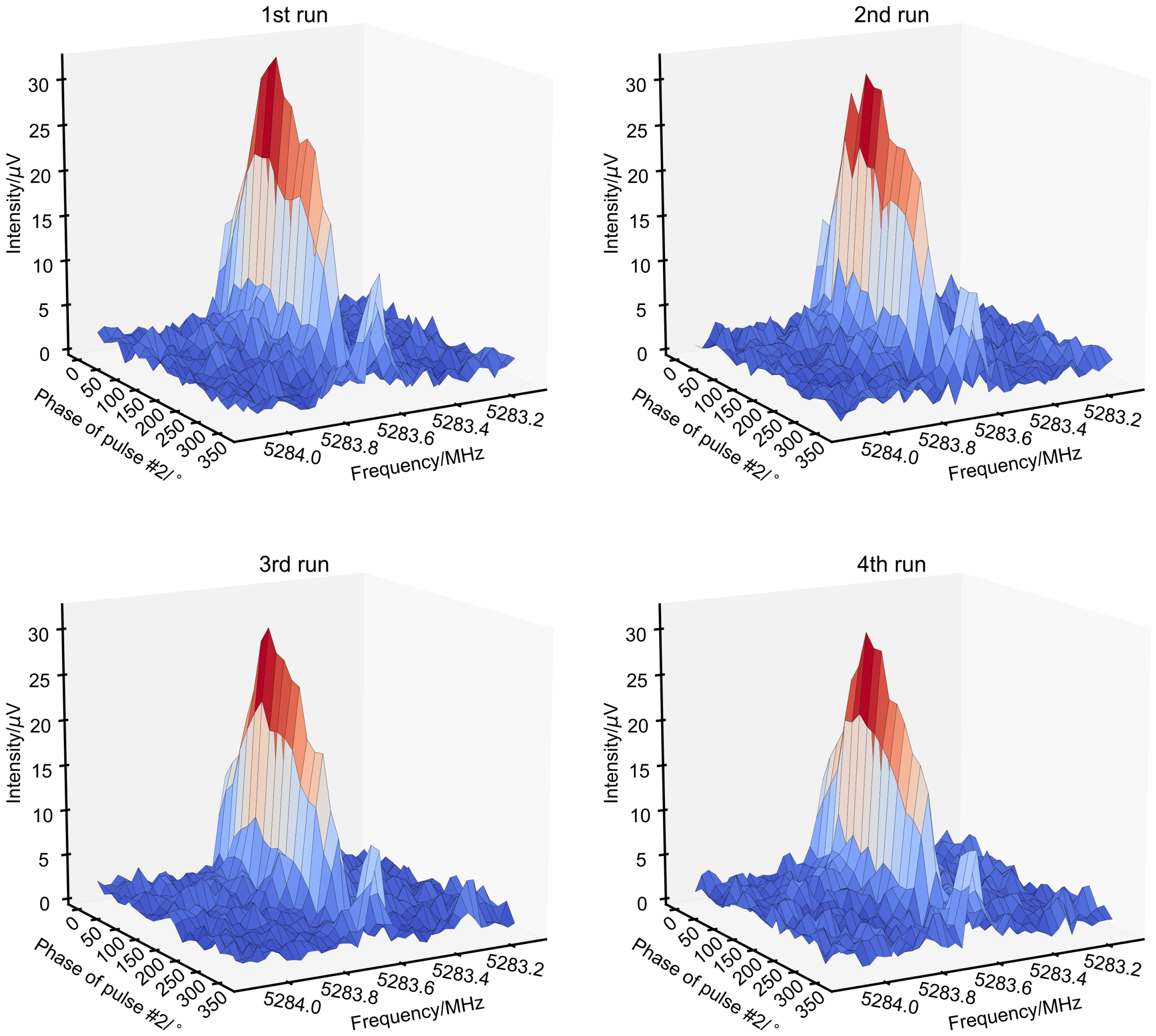

Supplementary Figure 11: 3D plot of the signal intensity of the listen transition at 5283.64 MHz while varying the phase of pulse #2, which corresponds to the $|2_{11}{}^{-}\rangle \rightarrow |2_{02}{}^{+}\rangle$ transition at 4373.14 MHz, in steps of 18°. The same experiment was performed four times continuously on the same day to ensure the reproducibility. Each step was averaged with 20,000 FIDs.

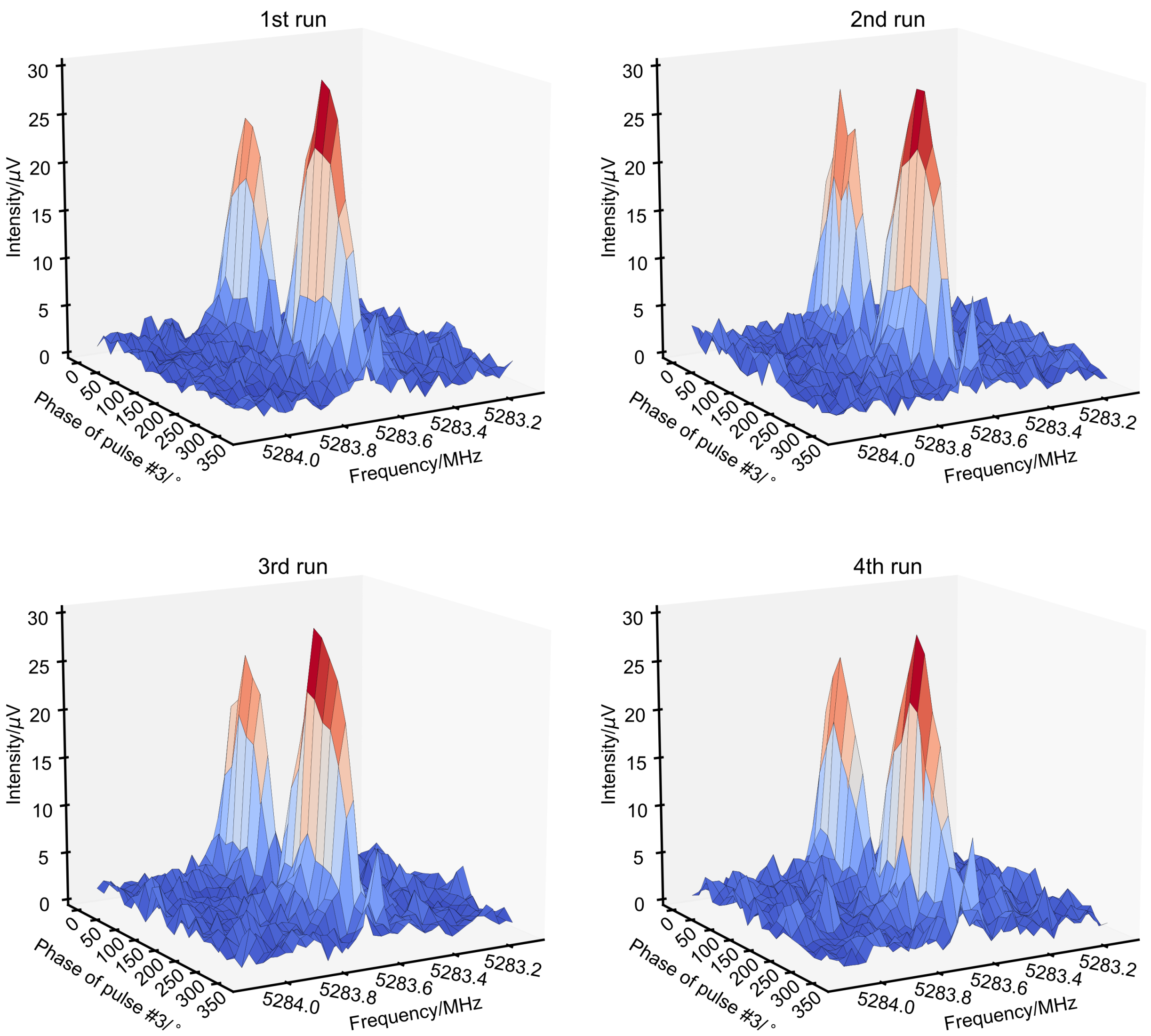

Supplementary Figure 12: 3D plot of the signal intensity of the listen transition at 5283.64 MHz while varying the phase of pulse #3, which corresponds to the $|2_{02}{}^{+}\rangle \rightarrow |1_{01}{}^{+}\rangle$ transition at 5293.46 MHz, in steps of 18°. The same experiment was performed four times continuously on the same day to ensure the reproducibility. Each step was averaged with 20,000 FIDs.

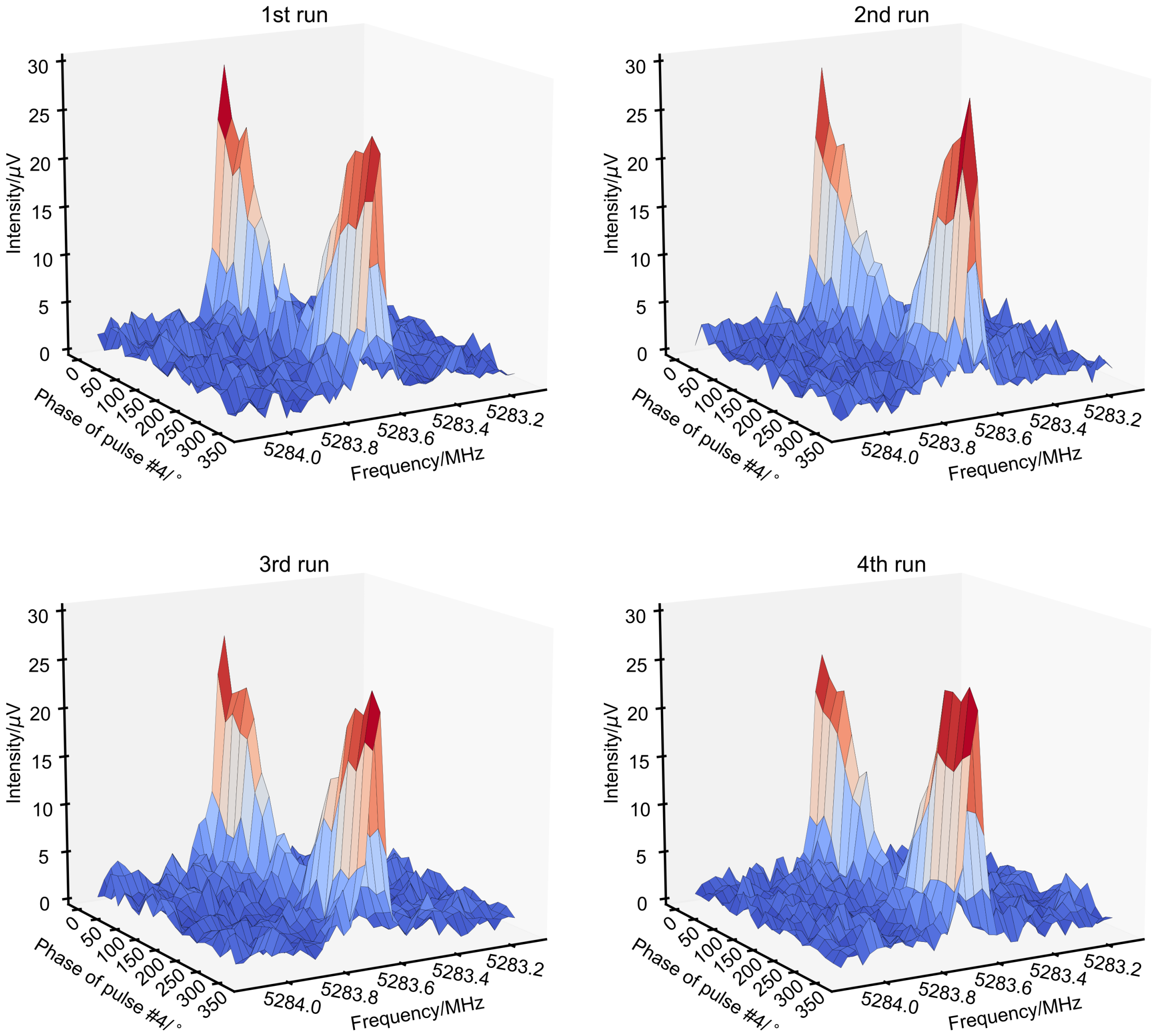

Supplementary Figure 13: 3D plot of the signal intensity of the listen transition at 5283.64 MHz while varying the phase of pulse #4, which corresponds to the $|2_{11}{}^{+}\rangle \rightarrow |1_{01}{}^{+}\rangle$ transition at 9230.54 MHz, in steps of 18°. The same experiment was performed four times continuously on the same day to ensure the reproducibility. Each step was averaged with 20,000 FIDs.

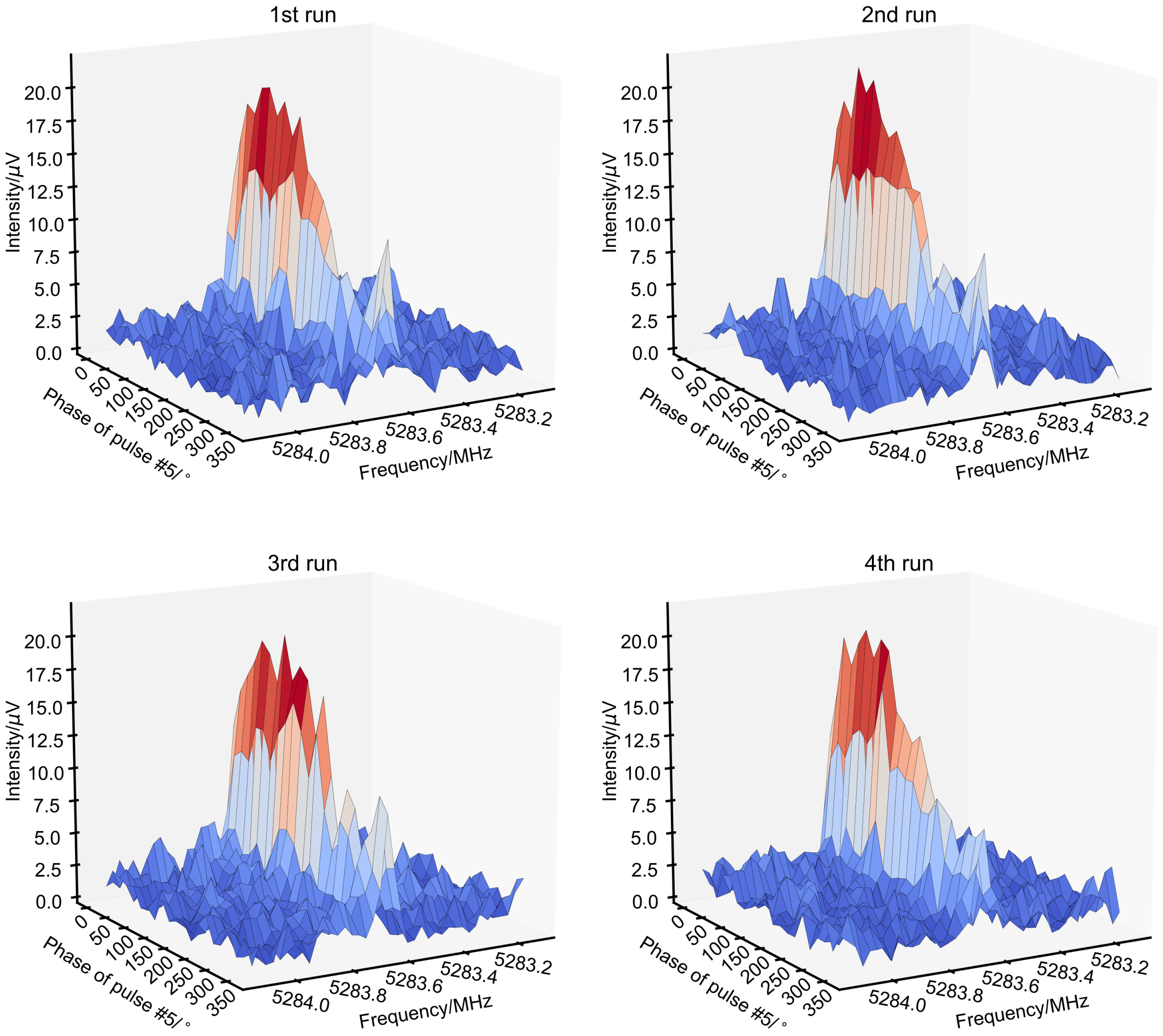

Supplementary Figure 14: 3D plot of the signal intensity of the listen transition at 5283.64 MHz while varying the phase of pulse #5, which corresponds to the $|2_{11}{}^{+}\rangle \rightarrow |2_{02}{}^{-}\rangle$ transition at 3458.65 MHz, in steps of 18°. The same experiment was performed four times continuously on the same day to ensure the reproducibility. Each step was averaged with 20,000 FIDs.

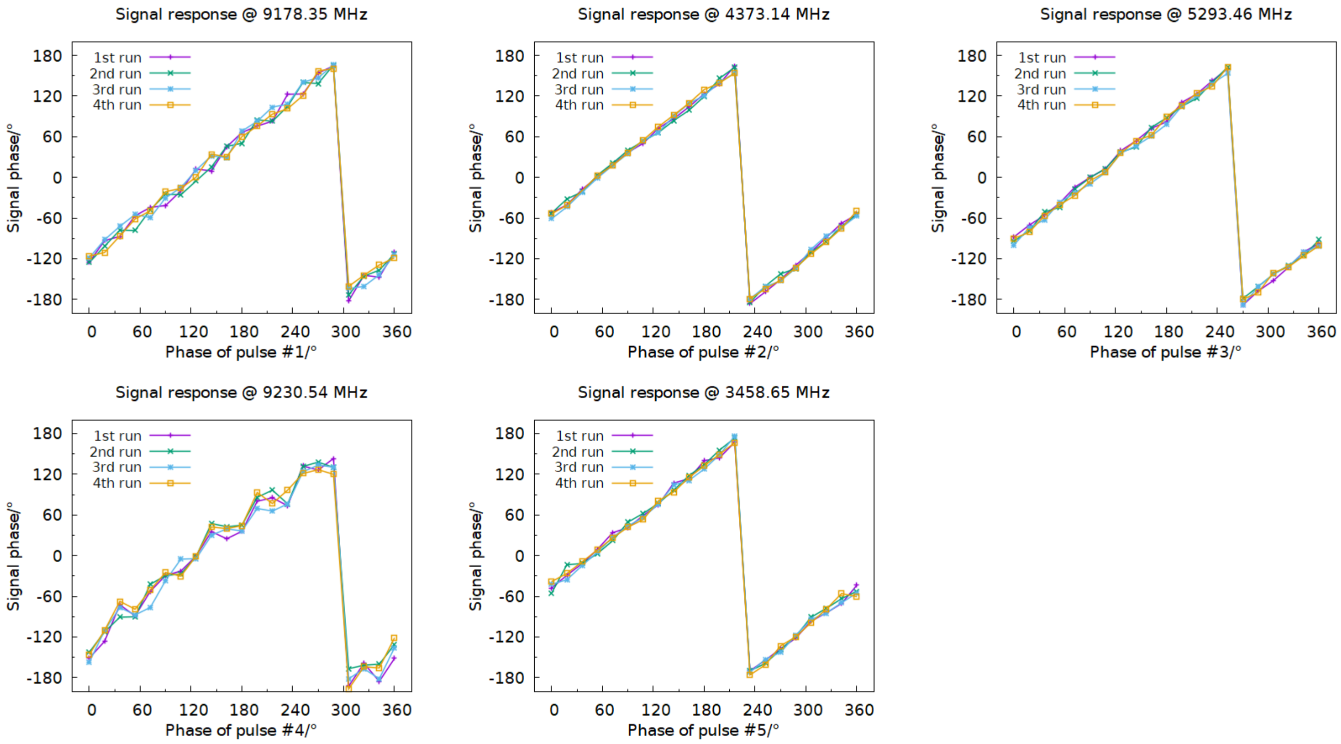

Supplementary Figure 15: Phase dependencies of the signal response at the scanned transition frequency in each set of the experiment, which shows a positively linear phase correlation between the excitation pulse and the molecular response at the same frequency.

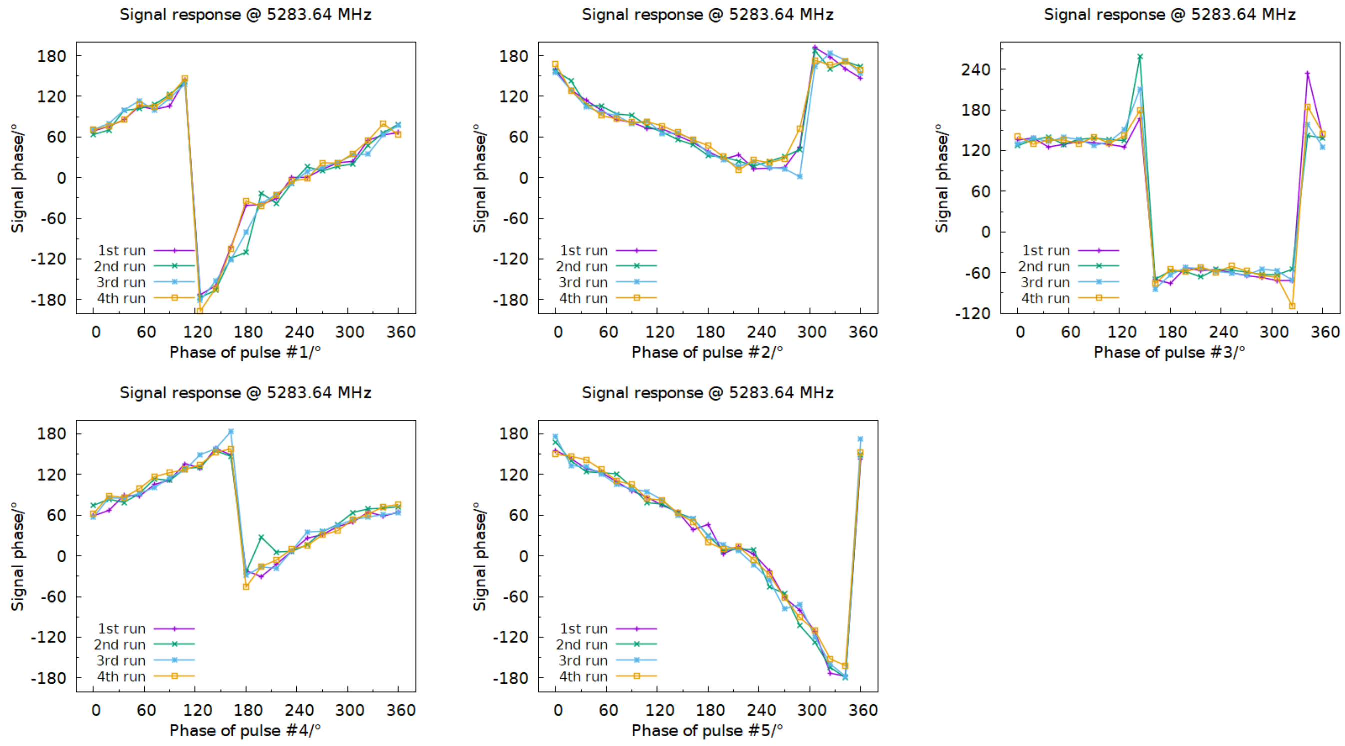

Supplementary Figure 16: Phase dependencies of the signal response at the listen transition in each set of the experiment, which shows the phase variation of the molecular response at the listen transition while scanning the phase of each excitation pulse of the scheme, as provided in Supplementary Figure 7 and Figure 1 in the main manuscript.

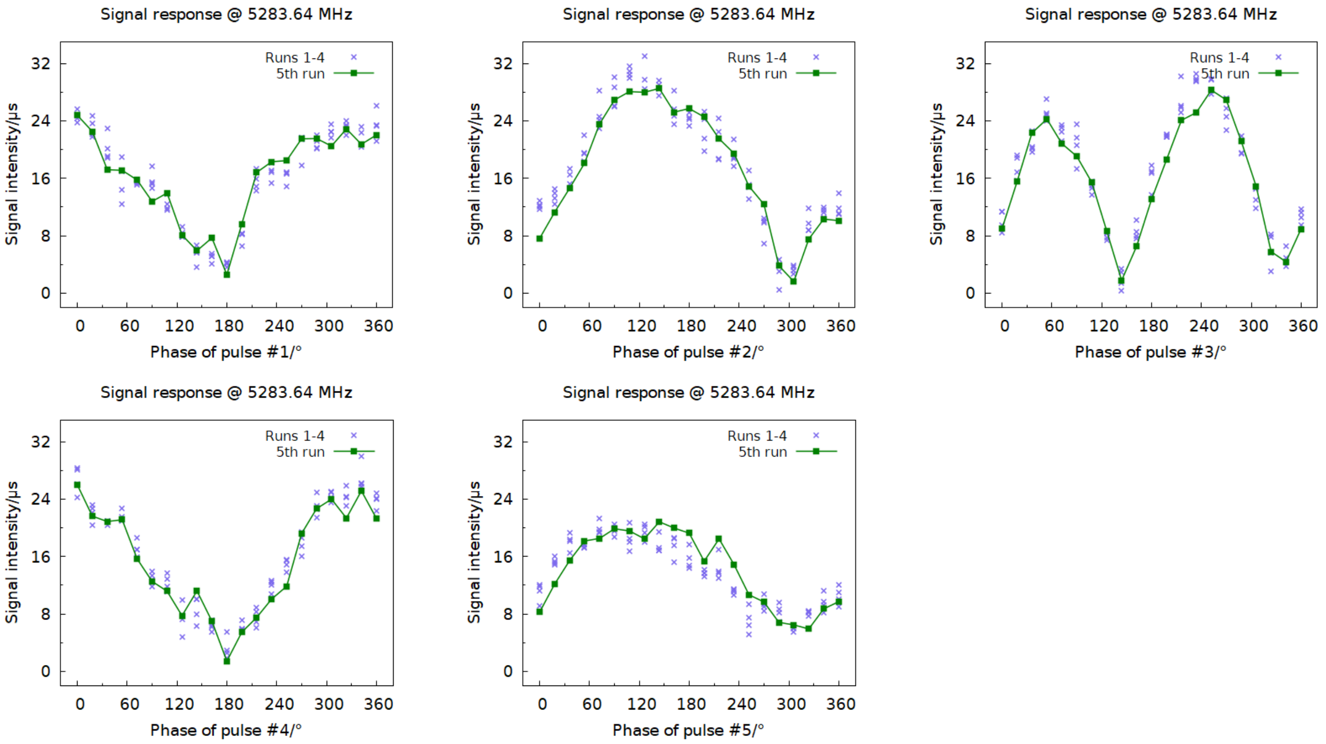

Supplementary Figure 17: Comparisons of the last repetition of the experiments with all four previous measurements. This shows a good reproducibility over the whole set of the experiments.

2.4 Testing of direct excitation by pulses from single amplifiers

During the experiment, although all five pulses are single-frequency waves and the listen transition frequency is not included, we noticed that the listen transition was co-excited due to spurious components produced by the amplifiers and the other components, which is a known phenomenon. The signal arising from a co-excitation could interfere with the molecular response induced from the M6WM cycle. Therefore, it is necessary to evaluate this in more detail. For this, the same pulse sequence was used as in the complete M6WM experiment unless otherwise stated. In the first set of experiments, only one amplifier was turned on, while the others were off (see also the experimental scheme in Figure 2 of the main manuscript). This assured that only the pulses coming through the active amplifier were exciting the molecular ensemble. The spectra were collected individually with 20,000 FIDs in the same detection polarization as the M6WM experiment. An obvious co-excitation response ($\sim$10 μV) was detected when the 3W SSA was on, as shown in panel A of Supplementary Figure 18, which was used to amplify pulse #3 (5293.46 MHz) in the sequence. To ensure no contribution from the other amplifiers, more averages (60,000) of the FIDs were recorded to increase the signal to noise ratio. The results for the 40 W SSA showed no evidence of co-excitation from pulses #1 and #4. However, for the 50 W SSA, which amplified pulses #2 and #5, a weak signal of $\sim$3 μV was observed. To find out which pulse causes the co-excitation, we removed one pulse (#2 or #5) from the sequence while collecting the spectrum with the other one. It turned out that the co-excitation came from pulse #2, as shown in panel B of Supplementary Figure 18. Eventually, pulses #2 and #3 were identified as two potential sources to cause co-excitations and interferences. The phases of the co-excited signals are linearly and positively dependent on the phases of pulses #2 and #3, correspondingly, as shown in Supplementary Figure 19.

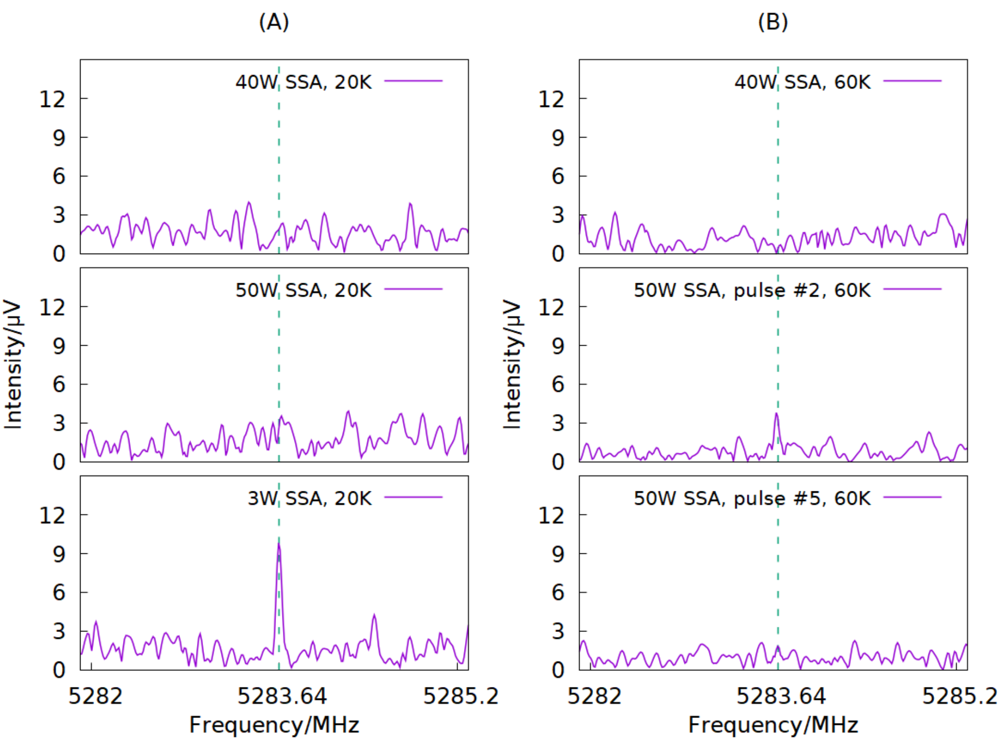

Supplementary Figure 18: Results from the amplifier tests. Panel A shows the spectra from an average of 20,000 FIDs with only one amplifier turned on. Panel B shows the spectra with 60,000 FIDs, where the top trace is with 40 W SSA and the other two are with 50 W SSA. When measuring the middle spectrum, pulse #5 was removed from the sequence, whereas pulse #2 was removed for the bottom spectrum.

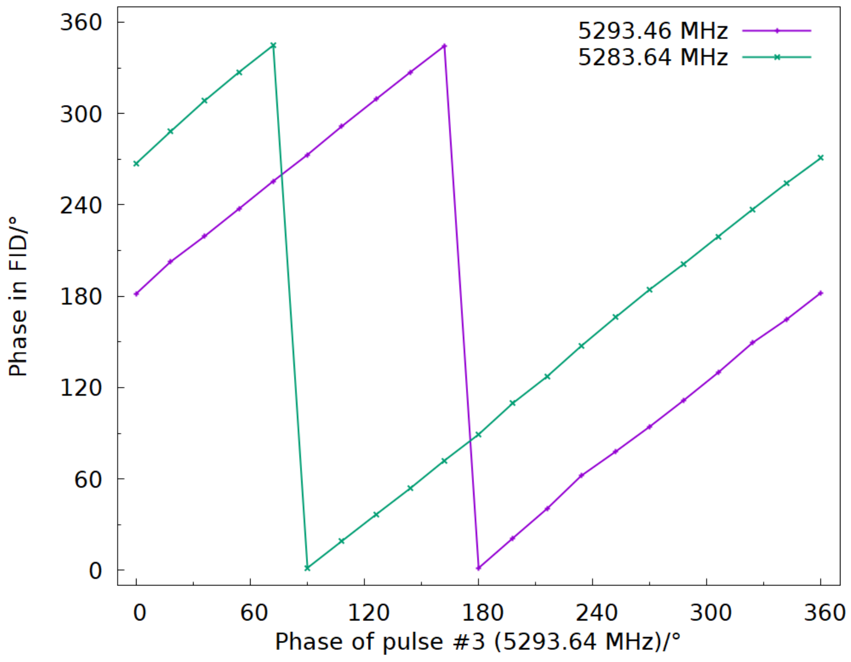

Supplementary Figure 19: Phase of the direct-excitation signal at 5293.46 MHz and the co-excited signal at 5283.64 MHz in the collected FID, when varying the phase of pulse #3 (5293.46 MHz) in the pulse sequence.

3 Supplementary data analysis

3.1 Windowed Fourier Transformation

To extract the intensity and the phase for a specific frequency from the free induction decay (FID) of the molecular ensemble, one can use the windowed Fourier Transformation.[25] The FID is given as a function of time (t), i.e., FID = FID(t). To get these scalar dependencies of the amplitude and phase of the signal at the listen frequency, we are performing the Fourier transformation ($\hat{\mathfrak{F}}$) with a filter function in the frequency domain, $F(\nu)$ (ν is the normal frequency), followed by averaging in the frequency domain:

$$S_{\nu_0} = \int_0^{+\infty} F(\nu) \cdot \underbrace{\left(\int_0^{+\infty} \mathrm{FID}(t) \cdot \exp(-2\pi i \nu t) dt \right)}_{\hat{\mathfrak{F}}(\nu)} d\nu =$$

$$= \int_0^{+\infty} \mathrm{FID}(t) \cdot \underbrace{\left(\int_0^{+\infty} F(\nu) \cdot \exp(-2\pi i \nu t) d\nu \right)}_{f(t)} dt = \int_0^{+\infty} \mathrm{FID}(t) \cdot f(t) dt \ , \quad (6)$$

here $f(t)$ is the time-domain image of the frequency domain filter, and $S_{\nu_0} = I_{\nu_0} \exp(i\varphi)$ is the result with intensity $I_{\nu_0} = |S_{\nu_0}| \in \mathbb{R}$ and the phase $\phi \in [-180°; 180) \subset \mathbb{R}$. The normalization condition for the filter is that

$$\int_0^{+\infty} F(\nu) d\nu = 1 \ .$$

3.1.1 Gaussian filter

The filter we applied is the Gaussian window:

$$F(\nu) = \frac{1}{\sigma\sqrt{2\pi}} \exp\left(-\frac{(\nu - \nu_0)^2}{2\sigma^2} \right) \ ,$$

where ν_0 is the central frequency of the window and σ is the filter width related to the full width at half maximum (FWHM) as FWHM $= 2\sqrt{2\ln(2)} \cdot \sigma$. The image of this filter is

$$f(t) = \int_0^{+\infty} F(\nu) \cdot \exp(-2\pi i \nu t) d\nu \approx \exp\left(-2\pi i \nu_0 t - \frac{(2\pi\sigma)^2}{2} t^2 \right) \ .$$

3.1.2 Rectangular filter

The alternative filter is the rectangular window:

$$F(\nu) = \begin{cases} 1/\text{FWHM} & , \text{ if } (\nu_0 - \text{FWHM}/2) \leq \nu \leq (\nu_0 + \text{FWHM}/2); \\ 0 & , \text{ otherwise} \end{cases} .$$

The image of this filter in the time domain is:

$$f(t) = \int_0^{+\infty} F(\nu) \cdot \exp(-2\pi i \nu t) d\nu = \exp(-2\pi i \nu_0 t) \cdot \text{sinc}(\pi \cdot \text{FWHM} \cdot t) ,$$

where $\text{sinc}(x) = \sin(x)/x$.

3.1.3 Computational procedure

A script implementing this procedure is given in Supplementary Section 3.3. Here, we briefly describe its algorithm. The actual calculation is done using the function `processSingleFile`.

1. The data is loaded from the file using the `numpy.loadfile` routine. The resulting data set is a two-dimensional array, where the rows are the time steps, the first column is the time (in seconds), and the second is the intensity (in mV).

2. The actual time 1D NumPy array (`t`) goes from `t`=0 to the total FID duration, and it is calculated in μs, to match multiplication to frequencies given in MHz.

3. The complex result of the integration according to Equation 6 is given as

$$S_{\nu_0} = \frac{1}{\text{HC}} \sum_{n=0}^{n<N} \text{FID}(t_n) \cdot f(t_n) ,$$

   where n enumerates the time steps, N is the total amount of points in the FID, and $t_n = \Delta t \cdot n$, where Δt is the time step calculated from the first column of the FID as $t_1 - t_0$, and the HC is the heuristic coefficient to match the scope-observed intensities (taken as 100 by default).

3.1.4 Script for the analysis

```
import argparse
import numpy as np
```

```python
import os

# function to parse the file names
# requires:
#           fName (str) -- a pathway to a file for processing
# returns:
#           a dictionary with the experiment parameters parsed from the file
#              with the following keys:
#                  - "duration"  -- duration of the pulse in us
#                  - "num_of_av" -- number of averages
#                  - "pulse_phase"     -- pulse phase in degrees
#                  - "base"      -- base name
def parseFileName(fName):
    actualFileName = fName.split(os.sep)[-1]
    actualFileName = actualFileName[:-4]
    word = actualFileName.split("_")

    parsedData = dict()

    if len(word) < 4:
        print("The file name: "+actualFileName+" does not look as:")
        print("   basename_phase_duration(of the pulse scanned)_#ofavg")
        raise RuntimeError("wrong file name format")

    parsedData["base"] = word[0]
    parsedData["pulse_phase"] = float(word[1][:-3])
    parsedData["duration"] = float(word[2][:-2])
    parsedData["num_of_av"] = int(word[3])

    return parsedData

# Function to process a single file
# requires:
#           fName (str) -- a pathway to a file for processing
#           v0 (float) -- central frequency
#           fwhm (float) -- FWHM of the filter
#           nrows2skip (int) -- number of the rows to skip in the file
# returns:
```

```
#            absolute average intensity in the window (float, in mV)
#            averga phase of the signal in the window (in degrees)
def processSingleFile(fName, v0, fwhm, nrows2skip, debug=False, doNotParseFileName=
    False, useRectWindow=False, heuristicCoef=1.0):

    if doNotParseFileName:
        finalDict = {"filename":fName}
    else:
        finalDict = parseFileName(fName)

    # data is in the format: time (in seconds) -- intensity (in mV)
    data = np.loadtxt(fName, dtype=float, skiprows=nrows2skip)
    dt = data[1][0] - data[0][0]  # time step in seconds

    if debug:
        print("Processing file %s , dt = %15.10f ns" % (fName, dt*1.e9) )

    t = 1.0e6*dt*np.arange(0,data.shape[0],dtype=float) # array of FID times, in
    microseconds (to be compatible with the MHz)
    width = 2.0*np.pi* fwhm/2.35482004503094938202 # this is the s (width) of the
    Gaussian defined as exp(-0.5*x**2/s**2) with x being v-v0 (normal, not angular
    frequencies)

    if not useRectWindow:  # Use the Gaussian Window
        # result is the integral of FID * exp(-i * 2*pi * v0 * t - 0.5 * w**2 * t
    **2)
        res = np.sum( data[:,1] * np.exp( -2j*np.pi*v0*t - 0.5*width**2 * t**2) )
        # norm is dt/(sqrt(2*pi)*sigma)
        finalDict["filter"] = "Gaussian"
    else:
        # result is the integral of FID * sinc(2 * pi * FWHM * t)
        res = np.sum( data[:,1] * np.exp( -2j*np.pi*v0*t) * np.sinc(fwhm * t) )
        finalDict["filter"] = "Rectangular"

    res /= heuristicCoef

    finalDict["signal_intensity"] = np.abs(res)
    finalDict["signal_phase"] = np.angle(res, deg=True)
```

```python

    finalDict["frequency"] = v0
    finalDict["fwhm"] = fwhm

    return finalDict

# Function to print the base of the output file
def printBaseOfTheOutput(output, doNotParseFileName):

    line = ""
    if doNotParseFileName:
        line = "# Frequency[MHz] SignalIntensity[mV]    SignalPhase[deg]  FWHM[MHz]  FileName   Filter"
    else:
        line = "# Frequency[MHz] PulsePhase[deg]  SignalIntensity[mV]  FWHM[MHz]  SignalPhase[deg]   PulseDuration[us]    NumOfAverages    FileNameBase   Filter"

    if output is None:
        print(line)
    else:
        output.write(line+"\n")
        output.flush()

# Function to print the results
# Requires:
#           res (dict) -- result of the processing of the file
#           output (None or open(file) ) -- thing to regulate/dump the results
def printResult(res, output, doNotParseFileName):

    line = ""
    if doNotParseFileName:
        line += " %15.10f " % (res["frequency"])
        line += " %15.10f " % (res["signal_intensity"])
        line += " %15.10f " % (res["signal_phase"])
        line += " %15.10f " % (res["fwhm"])
        line += " " + res["filename"]
        line += " " + res["filter"]
    else:
```

```
        line += " %15.10f " % (res["frequency"])
        line += " %7.3f " % (res["pulse_phase"])
        line += " %15.10f " % (res["signal_intensity"])
        line += " %15.10f " % (res["signal_phase"])
        line += " %15.10f " % (res["fwhm"])
        line += " %7.3f " % (res["duration"])
        line += " %7i " % (res["num_of_av"])
        line += " %s " % (res["base"])
        line += " " + res["filter"]

    if output is None:
        print(line)
    else:
        output.write(line+"\n")
        output.flush()

parser = argparse.ArgumentParser(description="Script to perform Fourier
    transformation of the FID trace with an averaged Gaussian-shaped window.")

parser.add_argument("-f", "--filename",
                    help="Filename of a single file to do the processing of",
                    type=str, default = None)

parser.add_argument("-p", "--path",
                    help="Path to a directory with multiple files to process",
                    type=str, default = None)

parser.add_argument("-v", "--frequency",
                    help="Center frequency of the window, in MHz",
                    type=float, default = None)

parser.add_argument("-w", "--FWHM",
                    help="FWHM of the Gaussian window, in MHz",
                    type=float, default = 1.0)

parser.add_argument("--NumOfRowsToSkip",
                    help="Number of rows to skip in the FID trace files",
                    type=int, default = 6)
```

```
parser.add_argument('--DebugMode', action='store_true',
                    help="Flag to print different shit during processing")

parser.add_argument('--NotParseFileNames', action='store_true',
                    help="Flag to stop trying to parse the file names")

parser.add_argument('--UseRectFilter', action='store_true',
                    help="Flag to switch Gaussian-shaped window to a rectangular window")


parser.add_argument("-c", "--HeuristicCoeff",
                    help="Heuristic coefficient to scale the initensity to the value observed on the scope",
                    type=float, default = 100.0)

parser.add_argument("-o", "--output",
                    help="Output file name",
                    type=str, default = None)

args,unknown = parser.parse_known_args() # parse arguments


# this is the output of the programm
if args.output is None:
    output = None
else:
    output = open(args.output, "w")

if not args.filename is None:
    res = processSingleFile(args.filename, v0=args.frequency, fwhm=args.FWHM, nrows2skip = args.NumOfRowsToSkip, debug=args.DebugMode, doNotParseFileName=args.NotParseFileNames, useRectWindow=args.UseRectFilter, heuristicCoef=args.HeuristicCoeff)
    printBaseOfTheOutput(output, args.NotParseFileNames)
    printResult(res, output, args.NotParseFileNames)

```

```python
elif not args.path is None:
    filelist = []
    for f in os.listdir(args.path):
        if f[-4:] == ".txt":
            filelist.append(f)

    filelist.sort(key=lambda x: os.path.getmtime(os.path.join(args.path, x)))

    if args.DebugMode:
        print(filelist)

    printBaseOfTheOutput(output, args.NotParseFileNames)

    for f in filelist:
        res = processSingleFile(os.path.join(args.path, f), v0=args.frequency, fwhm=args.FWHM, nrows2skip = args.NumOfRowsToSkip, debug=args.DebugMode, doNotParseFileName=args.NotParseFileNames, useRectWindow=args.UseRectFilter, heuristicCoef=args.HeuristicCoeff)
        printResult(res, output, args.NotParseFileNames)
```

3.2 Description of the interference patterns

3.2.1 General formulas

The experimental signal generated from the cycle given in Supplementary Figure 2 turned out to be mixed with the direct excitations $|0-\rangle \to |2-\rangle$ coming from some of the pulses within the sequence, as described in Supplementary Section 2.4. This results in interference patterns during the phase scans for the different pulses. Here, we will give a brief description of how those were treated.

In general, each of our signals can be represented as a sine wave (see e.g. Equation 3):

$$S_k(t) = a_k \cdot \sin(\omega t + \overbrace{s_k \varphi + \phi_k}^{\theta_k}) \ ,$$

where ω is the listen transition frequency, a_k is the amplitude of the k-th signal, ϕ_k is the starting amplitude of the signal, given by the different state oscillation terms $\exp(-iEt/\hbar)$, φ is the scanned phase of the electric field, and $s = 0, \pm 1$ is the coefficient determining whether the electric field

- caused absorption, i.e., going up in energy within the cycle ($s = +1$),

- did not affect this signal ($s = 0$),

- caused emission, i.e., going down in energy within the cycle ($s = -1$).

A signal composed of N contributions will thus be:

$$S(t) = \sum_{k=1}^{N} S_k(t) = A \cdot \sin(\omega t + \Phi) \ ,$$

where the total amplitude of the signal (A) and total phase (Φ), extracted from the Fourier transformation (see Supplementary Section 3.1), depend on the parameters of each k-th contribution. This sum can be computed analytically. Since $\sin(z_1 + z_2) = \sin(z_1)\cos(z_2) + \cos(z_1)\sin(z_2)$,[26] one can rewrite $S(t)$ as

$$S(t) = \sum_{k=1}^{N} a_k \cdot [\sin(\omega t)\cos(\theta_k) + \cos(\omega t)\sin(\theta_k)] = \sin(\omega t) \cdot \underbrace{\left[\sum_{k=1}^{N} a_k \cos(\theta_k)\right]}_{\alpha} + \cos(\omega t) \cdot \underbrace{\left[\sum_{k=1}^{N} a_k \sin(\theta_k)\right]}_{\beta} \ .$$

At the same time, $S(t) = A \cdot [\sin(\omega t)\cos(\Phi) + \cos(\omega t)\sin(\Phi)]$, and thus

$$\begin{cases} \alpha = \sum_{k=1}^{N} a_k \cos(\theta_k) = A \cdot \cos(\Phi) \ , \\ \beta = \sum_{k=1}^{N} a_k \sin(\theta_k) = A \cdot \sin(\Phi) \ . \end{cases}$$

Thus,

$$\tan(\Phi) = \frac{\beta}{\alpha} = \frac{\sum_{k=1}^{N} a_k \sin(s_k\varphi + \phi_k)}{\sum_{k=1}^{N} a_k \cos(s_k\varphi + \phi_k)} \ , \tag{7}$$

and[26]

$$A^2 = \alpha^2 + \beta^2 = \sum_{k=1}^{N}\sum_{l=1}^{N} a_k a_l \cdot \overbrace{[\cos(\theta_k)\cos(\theta_l) + \sin(\theta_k)\sin(\theta_l)]}^{\cos(\theta_k - \theta_l)} = \sum_{k=1}^{N}\sum_{l=1}^{N} a_k a_l \cos(s_k\varphi + \phi_k - s_l\varphi - \phi_l) \ . \tag{8}$$

These formulas were used for fitting the interference patterns (see Supplementary Section 3.3).

3.2.2 The case of two interfering signals

The simplest case refers to two interfering signals. Let us assume that the signal of the $|2-\rangle \rightarrow |0-\rangle$ transition (Supplementary Figure 4) is composed of two components:

- one is the signal from the cycle given in Supplementary Figure 2

$$S_5(t) = A_5 \cdot \sin(\omega t + \sum_{k=1}^{5} s_{5,k}\varphi_k + \phi_5) \ ,$$

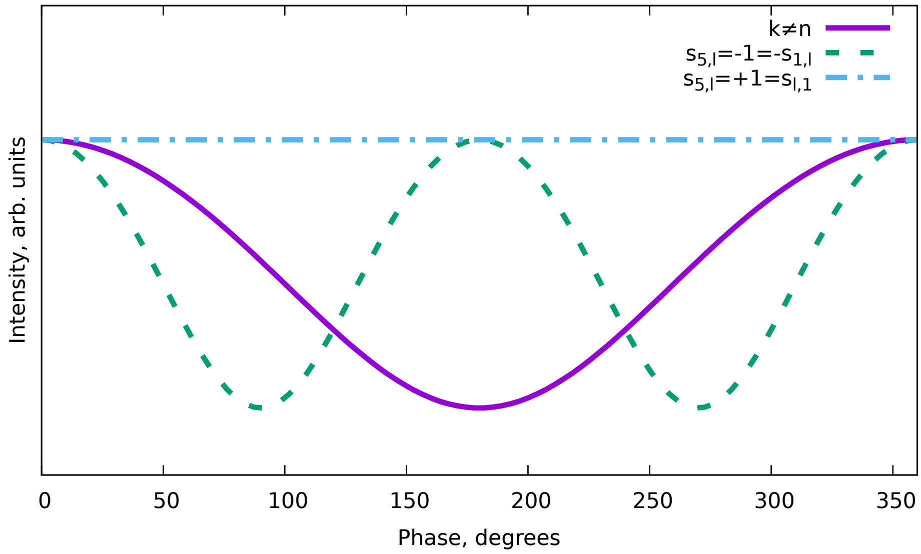

Supplementary Figure 20: Three basic shapes of expected interference patterns.

- the second arises from the direct excitation of the listen transition ($|0-\rangle \rightarrow |2-\rangle$) by one ($l$) of the excitation pulses ($l \in [1; 5]$)

$$S_1(t) = A_1 \cdot \sin(\omega t + s_{1,l}\varphi_l + \phi_1) \ .$$

$S_5(t)$ depends on the phases of all five pulses ($\{\varphi_k\}_{k=1}^5$) with $s_{5,k} = \pm 1$, whilst the other signal depends on the phase of only one of the pulses, φ_l, and it corresponds only to $|0-\rangle \rightarrow |2-\rangle$ absorption, i.e. $s_{1,l} = +1$. This gives three possible scenarios for the interference (Supplementary Figure 20).

- If the φ_k is scanned, for which $k \neq l$ (i.e. the scanned pulse does not cause direct excitation), then the amplitude is given as

$$A(\varphi_k) = \sqrt{A_5^2 + A_1^2 + 2A_5A_1 \cdot \cos(s_{5,k}\varphi_k + \ldots)} \ ,$$

which corresponds to a function with one minimum.

- If $k = l$ (the scanned pulse causes also the interfering direct excitation), two more cases are possible:
  - $s_{5,k} = -1 = -s_{1,k}$ (i.e., in the cycle the pulse #k causes emission). In this case the amplitude variation would have two minima:

$$A(\varphi_k) = \sqrt{A_5^2 + A_1^2 + 2A_5A_1 \cdot \cos(2 \cdot \varphi_k + \ldots)} \ .$$

  - $s_{5,k} = +1 = s_{1,k}$ (i.e., in the cycle the pulse #k causes absorption, as in $S_1(t)$). In this case the

amplitude would not depend on the phase φ_l, but would be constant:

$$A(\varphi_k) = \sqrt{A_5^2 + A_1^2 + 2A_5A_1 \cdot \cos(0 \cdot \varphi_k + \ldots)}\ .$$

3.2.3 The case of more than two interfering signals

The variety of the interference patterns of the amplitudes appearing during the pulse phase scans in the case of more than two pulses is significant. However, in some cases they simplify themselves. Let us assume that we scan a phase φ_k, and we have multiple signals $S_l(t) = A_l \cdot \sin(\omega t + \tilde{\phi}_l)$ with their phases $\tilde{\phi}_l$ independent of φ_k. In this case, they will combine to a single effective phase-independent signal $S_{\text{eff}}(t) = A \cdot \sin(\omega t + \tilde{\Phi})$, where A and $\tilde{\Phi}$ are given with Equations 7 and 8.

3.3 Fitting of the interference patterns

Theoretical details on the interference patterns are given in Supplementary Section 3.2. Here, we provide a script used to perform the fitting. The interference signal is thought to be a sum of N signals $\{S_k\}_{k=1}^N$, that are given by their intensity A_k, offset phase ϕ_k, and the signature s_k:

$$S_k = A_k \cdot \sin(s_k \cdot \varphi + \phi_k)\ ,$$

where φ is the scanned phase. The least-squares functional used for the minimization is

$$F(\boldsymbol{\xi}) = \sum_{i=1}^{M} \left(\frac{1}{\delta A^2} \cdot (A_i^{(\text{theor})}(\boldsymbol{\xi}) - A_i^{(\text{exp})})^2 + \frac{0.1}{\delta t P^2} (tP_i^{(\text{theor})}(\boldsymbol{\xi}) - tP_i^{(\text{exp})})^2 \right)\ ,$$

where $\boldsymbol{\xi}$ denotes a set of signal parameters $\{A_k, \phi_k\}_{k=1}^N$, i enumerates experimental data points, M is the total number of experimental points, “theor” and “exp” indexes indicate theoretical and experimental values, tP is the tangent of the phase ($tP = \tan(\Phi)$), and $\delta X = \max(X^{(\text{exp})}) - \min(X^{(\text{exp})})$. Fitting requires only the experimental points, and the set of signal signatures $\{s_k\}_{k=1}^N$.

```
import numpy as np
import sys
import random as rnd
import scipy.optimize as spo

#####
deg2rad = np.pi/180.0
```

```
#####

##### General idea of the algorithm
#####    1. Do the global fit of the signal amplitude using the following parameters:
#####                     A = Atheor(amplitudes of the signals, phases of all the signals)
#####    2. Do the global fit of the signal phase using the following parameters:
#####                     Phase = Phase_theor(phase shift of all the signals, phase shift of between theory and the experiment)
#####                                 i.e. relative phases of the individual contributions, as well as the amplitudes of the signals are frozen
#####         2.1 fit the phase of the signal as Pexp = Pshif  + Ptheor(Pint_shift)
#####         2.2 fit the phase of the signal as Pexp = Pshif  - Ptheor(Pint_shift)
#####       The final result is the minimal choice between results of 2.1 and 2.2


# this function returns the theoretical phase of the signals
# x is the pulse phases, in degrees (np.array of floats)
# A is the list of amplitudes (floats)
# Ph is the list of constan phases (floats in range of [-pi:pi])
# N is the list of the coefficients (ints)
def phase(x, Amplitudes, Phases, N):
    try:
        sPhi = np.zeros(x.shape)
        cPhi = np.zeros(x.shape)
    except:
        sPhi = 0.0
        cPhi = 0.0
    for i,a in enumerate(Amplitudes):
        sPhi += a * np.sin(deg2rad * (N[i] * x + Phases[i]) )
        cPhi += a * np.cos(deg2rad * (N[i] * x + Phases[i]) )
    thr = 1.0e-30

    cPhi =  np.where( np.abs(cPhi)> thr, cPhi, thr  )
    tgPhi = sPhi/cPhi
    return tgPhi

```

```python
# returns squared amplitude
def amplitude(x, A, Ph, N):
    try:
        amp2 = np.zeros(x.shape)
    except:
        amp2 = 0.0

    for i,ai in enumerate(A):
        for j,aj in enumerate(A):
            amp2 += ai*aj * np.cos(deg2rad * (N[i] * x + Ph[i] - N[j] * x - Ph[j]) )

    return np.sqrt(amp2)

def ConvSignatureToName(N):
    res = ""
    for i,n in enumerate(N):
        if i>0:
            res += "_"
        res += "%i" % (n)
    return res

def SplitSignalParamsVector(x, NumOfSignals):
    SignalAmp   = x[:NumOfSignals]
    SignalPhase = x[NumOfSignals:]
    return SignalAmp,SignalPhase


def PhaseLSQMetric(y1, y2):
    possibleShifts = (0.0, -360., +360.)
    if np.isscalar(y1):
        d = np.abs(y1 - y2)
        for s1 in  possibleShifts:
            for s2 in possibleShifts:
                td = np.abs( (y1 + s1) - (y2 + s2) )
                if td < d:
                    d = td
        return d**2
    else:
```

```python
        return np.array([PhaseLSQMetric(y1[i], y2[i]) for i in range(0,len(y1))])

if __name__ == "__main__":


    ###### Step 0: read input information
    if len(sys.argv)<2:
        print("Nothing to process")
        exit()
    else:
        print("#Processing file "+sys.argv[1])

    data = np.loadtxt(sys.argv[1],  usecols=(1,2,3), dtype=float)

    SignalNCoeffs = np.array([1])

    if len(sys.argv)>3:
        SignalNCoeffs = np.array([int(a) for a in sys.argv[2:] ])

    NSamples = 1000
    DoGnuplot = True

    NumOfSignals = len(SignalNCoeffs)

    X      = data[:,0]
    YAmp   = data[:,1]
    YPhase = np.tan(data[:,2]*deg2rad)


    dAmp   = max(YAmp) - min(YAmp)
    dPhase = max(YPhase) - min(YPhase)

    fineGridX = np.linspace(min(X), max(X), NSamples)

    ####### Step 1: do the amplitude global fitting

    ## This is the function to be minimized in the fitting
```

```
 ## x -- is the concatenated array of the individual amplitues and the phases of the signal
 def AmplitudeAndPhaseLSQ(x):
     SignalAmp,SignalPhase = SplitSignalParamsVector(x = x , NumOfSignals = NumOfSignals)

     YtheorAmp   = amplitude(x = X, A = SignalAmp, Ph = SignalPhase, N = SignalNCoeffs)
     YtheorPhase = phase(x = X, Amplitudes = SignalAmp, Phases = SignalPhase, N = SignalNCoeffs)
     return np.sum( ((YAmp - YtheorAmp)/dAmp)**2 ) + 0.1 * np.sum( ((YPhase - YtheorPhase)/dPhase)**2 )


 maxAmp = max(YAmp)
 Bounds = tuple( [ (0.0, 5.0*maxAmp) for i in range(0,NumOfSignals)] + [(-180.,180.) for i in range(0,NumOfSignals)]  )

 res = spo.differential_evolution(func=AmplitudeAndPhaseLSQ, bounds=Bounds, maxiter=1000, popsize=50, seed=None)

 print("# Minimal LSQ for Amplitude fitting: " +str(AmplitudeAndPhaseLSQ(res.x)))
 paramLine = ""
 for tx in res.x:
     paramLine += " %f " % (tx)
 print("# Parameters : "+paramLine)

 FittedAmp,FittedPhase = SplitSignalParamsVector(x = res.x , NumOfSignals = NumOfSignals)

 ###### Step ?: print the results



 AddLine = ConvSignatureToName(N = SignalNCoeffs)
 fineGridYAmp = amplitude(x = fineGridX, A = FittedAmp, Ph = FittedPhase, N = SignalNCoeffs)
```

```
    fineGridYPhase = phase(x = fineGridX, Amplitudes = FittedAmp, Phases = FittedPhase, N = SignalNCoeffs)
    np.savetxt("fit_result_amplitude_"+sys.argv[1]+"_"+AddLine+"_.res", np.stack([fineGridX,fineGridYAmp],axis=-1))
    np.savetxt("fit_result_phase_"+sys.argv[1]+"_"+AddLine+"_.res", np.stack([fineGridX,fineGridYPhase],axis=-1))



    if DoGnuplot:
        line = "\n\n"
        for i in range(0,NumOfSignals):
            line += "a%i = %15.10f \n" % (i, FittedAmp[i])
            line += "p%i = %15.10f \n" % (i, FittedPhase[i])
            line += "n%i = %15.10f \n" % (i, SignalNCoeffs[i])

        line += "\nA(x) = sqrt("
        for i in range(0,NumOfSignals):
            if i>0:
                line += " + "
            line += " a%i**2 " % (i)
            for j in range(0,NumOfSignals):
                if i!=j:
                    line += " + a%i * a%i * cos( (pi/180.) * (n%i*x + p%i - n%i*x - p%i)  ) " % (i,j, i, i, j, j)
        line += ")\n\n"

        line += "\nP(x) = (180./pi) * atan("
        lineU = ""
        lineD = ""
        for i in range(0,NumOfSignals):
            if i>0:
                lineU += " + "
                lineD += " + "

            lineU += " a%i * sin( (pi/180.) * (n%i*x + p%i) ) " % (i,i,i)
            lineD += " a%i * cos( (pi/180.) * (n%i*x + p%i) ) " % (i,i,i)

```

```
        line += "("+lineU+") / (" +lineD + "))\n\n"

        print(line)

        print("set terminal pngcairo")
        print("set output '%s_amplitude.png" % (sys.argv[1]) )
        print("set xlabel 'Scanned phase, degrees'")
        print("set ylabel 'Amplitude, uV'")

        print("plot '%s' u 2:3 w lp title 'exp.', A(x) w l title 'fit'\n#pause -1" % sys.argv[1])

        print("set ylabel 'Singal phase, degrees'")
        print("set output '%s_phase.png" % (sys.argv[1]) )
        print("\n\nplot '%s' u 2:(180*atan(tan($4*pi/180))/pi) w lp title 'exp.', P(x) w l title 'fit'\n#pause -1" % sys.argv[1])
```

4 Supplementary experimental data

In this section, we will provide the experimental data and their fits. All the results were obtained with Gaussian-windowed Fourier transformation with the central frequency $\nu_0 = 5283.64$ MHz and the filter FWHM = 0.03 MHz.

4.1 Phase scan for pulse #1 (9178 MHz)

Supplementary Table 2: Amplitude (in mV) of the listen signal at 5283.64 MHz obtained from scanning the phase of the first pulse of the sequence given in Supplementary Figure 7 (9178 MHz).

```
# Intensity of the listen signal, mV
# Column #1 -- phase of the scanned pulse, in degrees
# Column #2 -- mean intensity for all measurements
# Column #3 -- standard deviation of the intensity for all measurements
# Columns from #4 and on -- the individually measured points
   0 0.0246 0.0006  0.0246 0.0257 0.0244 0.0237 0.0248
  18 0.0229 0.0011  0.0247 0.0221 0.0236 0.0217 0.0225
  36 0.0196 0.0019  0.0201 0.0229 0.0188 0.0191 0.0172
  54 0.0160 0.0023  0.0144 0.0124 0.0190 0.0170 0.0171
  72 0.0155 0.0002  0.0154 0.0156 0.0151 0.0155 0.0158
  90 0.0151 0.0016  0.0152 0.0176 0.0147 0.0154 0.0128
 108 0.0127 0.0010  0.0138 0.0115 0.0124 0.0119 0.0139
 126 0.0083 0.0005  0.0084 0.0078 0.0092 0.0078 0.0081
 144 0.0055 0.0010  0.0036 0.0058 0.0067 0.0056 0.0059
 162 0.0055 0.0012  0.0052 0.0041 0.0052 0.0055 0.0077
 180 0.0037 0.0006  0.0041 0.0036 0.0042 0.0043 0.0026
 198 0.0082 0.0010  0.0081 0.0065 0.0083 0.0083 0.0096
 216 0.0158 0.0011  0.0143 0.0149 0.0159 0.0173 0.0168
 234 0.0171 0.0011  0.0171 0.0181 0.0153 0.0169 0.0183
 252 0.0167 0.0012  0.0148 0.0169 0.0167 0.0166 0.0185
 270 0.0208 0.0015  0.0178 0.0217 0.0217 0.0214 0.0215
 288 0.0210 0.0007  0.0202 0.0212 0.0201 0.0220 0.0215
 306 0.0221 0.0010  0.0225 0.0225 0.0236 0.0217 0.0205
 324 0.0231 0.0007  0.0220 0.0232 0.0234 0.0240 0.0229
 342 0.0215 0.0011  0.0203 0.0232 0.0207 0.0223 0.0207
 360 0.0232 0.0017  0.0211 0.0233 0.0234 0.0260 0.0220
```

Supplementary Table 3: Phase (in degrees) of the listen signal at 5283.64 MHz obtained from scanning the phase of the first pulse of the sequence given in Supplementary Figure 7 (9178 MHz).

```
# Phase of the listen signal, degrees
# Column #1 -- phase of the scanned pulse, in degrees
# Column #2 -- mean phase for all measurements
# Column #3 -- standard deviation of the phase for all measurements
# Columns from #4 and on -- the individually measured points
  0    68.5    2.5    68.7    63.8    71.0    70.3    68.8
 18    75.2    3.1    75.9    70.1    79.7    76.3    74.1
 36    92.3   82.4    85.3    99.2    99.6    85.2    92.3
 54   106.0    4.5   105.4   101.6   113.7   107.7   101.5
 72   103.7    3.3   100.7   108.0    99.2   104.1   106.3
 90   115.9    6.0   105.6   122.6   117.4   120.4   113.6
108   144.7    5.4   143.9   141.0   138.1   146.4   154.0
126    31.2    9.9  -172.6  -177.0   178.7   163.3   163.7
144  -159.0    5.1  -159.4  -165.7  -152.3  -163.2  -154.4
162  -107.6   63.2  -103.0  -118.7  -121.3  -105.7   -89.3
180   -64.2   51.6   -41.1  -110.1   -80.8   -34.1   -54.8
198   -35.2    7.0   -39.6   -22.7   -38.5   -42.4   -32.8
216   -30.0    4.8   -30.6   -38.0   -24.1   -25.9   -31.5
234    -6.1    3.5     0.4    -8.4    -8.9    -5.1    -8.4
252     5.1    6.5     0.6    15.9     9.2    -1.5     1.4
270    16.5    5.1    12.3    10.0    15.5    21.9    22.9
288    22.0    3.6    21.9    16.7    22.3    21.4    27.9
306    29.1    5.9    24.1    20.3    33.1    35.3    32.9
324    48.5    7.3    55.1    47.7    34.9    54.7    50.4
342    69.4    6.7    63.0    66.4    63.2    79.6    74.9
360    70.1    6.7    66.7    79.0    77.4    63.0    64.4
```

Supplementary Table 4: Fitted parameters of the listen signal at 5283.64 MHz from scanning the phase of the first pulse of the sequence given in Supplementary Figure 7 (9178 MHz).

Signal no.	Signature (s)	Amplitude (A, mV)	Phase (ϕ, degrees)
1	0	0.0101355120	70.0000000000
2	1	0.0135546954	79.4452855239

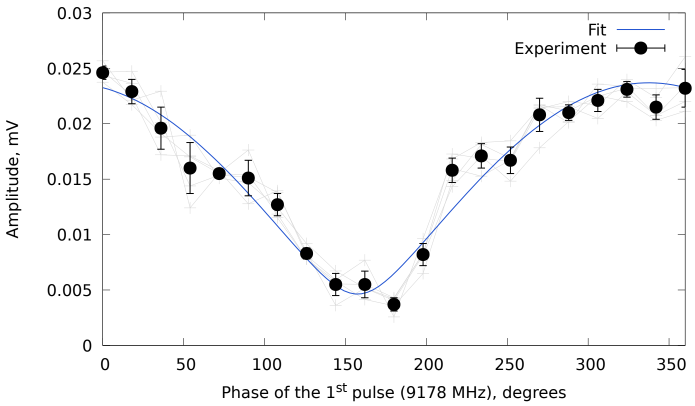

Supplementary Figure 21: The experimental amplitude of the listen signal at 5283.64 MHz obtained from scanning the phase of the first pulse of the sequence given in Supplementary Figure 7 (9178 MHz) and its fit. The circles with error bars are the values averaged over the individual measurements (given as gray lines with cross-shaped points). Solid blue line represents the fit result.

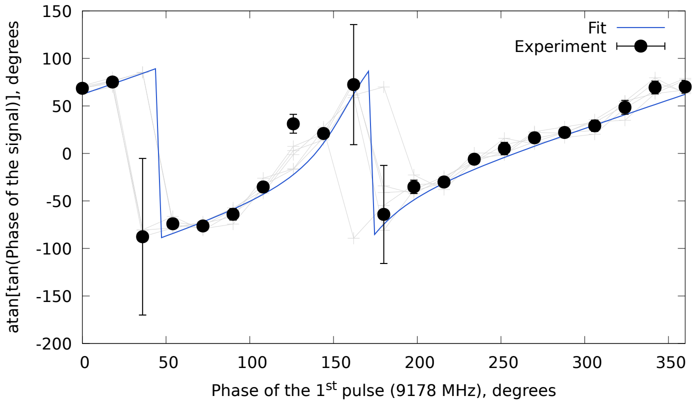

Supplementary Figure 22: The experimental phase of the listen signal at 5283.64 MHz obtained from scanning the phase of the first pulse of the sequence given in Supplementary Figure 7 (9178 MHz) and its fit. The circles with error bars are the values averaged over the individual measurements (given as gray lines with cross-shaped points). Solid blue line represents the fit result.

4.2 Phase scan for pulse #2 (4373 MHz)

Supplementary Table 5: Amplitude (in mV) of the listen signal at 5283.64 MHz obtained from scanning the phase of the second pulse of the sequence given in Supplementary Figure 7 (4373 MHz).

```
# Intensity of the listen signal, mV
# Column #1 -- phase of the scanned pulse, in degrees
# Column #2 -- mean intensity for all measurements
# Column #3 -- standard deviation of the intensity for all measurements
# Columns from #4 and on -- the individually measured points
   0 0.0113 0.0019  0.0123 0.0129 0.0121 0.0117 0.0076
  18 0.0131 0.0011  0.0132 0.0124 0.0139 0.0145 0.0113
  36 0.0159 0.0010  0.0165 0.0173 0.0152 0.0146
  54 0.0195 0.0014  0.0194 0.0220 0.0196 0.0183 0.0181
```

```
 72 0.0247 0.0019  0.0246 0.0282 0.0229 0.0241 0.0235
 90 0.0275 0.0016  0.0301 0.0260 0.0287 0.0259 0.0269
108 0.0302 0.0012  0.0316 0.0309 0.0304 0.0299 0.0281
126 0.0294 0.0019  0.0330 0.0297 0.0279 0.0285 0.0280
144 0.0286 0.0007  0.0290 0.0297 0.0275 0.0284 0.0285
162 0.0254 0.0015  0.0282 0.0247 0.0256 0.0235 0.0252
180 0.0246 0.0008  0.0244 0.0242 0.0252 0.0233 0.0257
198 0.0231 0.0021  0.0253 0.0242 0.0198 0.0215 0.0245
216 0.0211 0.0022  0.0243 0.0224 0.0188 0.0186 0.0215
234 0.0192 0.0012  0.0187 0.0214 0.0189 0.0176 0.0195
252 0.0150 0.0013  0.0171 0.0147 0.0131 0.0151 0.0149
270 0.0099 0.0018  0.0098 0.0101 0.0104 0.0069 0.0124
288 0.0032 0.0015  0.0031 0.0047 0.0004 0.0039 0.0038
306 0.0030 0.0008  0.0026 0.0036 0.0032 0.0039 0.0016
324 0.0093 0.0014  0.0087 0.0118 0.0088 0.0097 0.0075
342 0.0111 0.0006  0.0120 0.0116 0.0112 0.0106 0.0103
360 0.0116 0.0013  0.0139 0.0119 0.0110 0.0111 0.0101
```

Supplementary Table 6: Phase (in degrees) of the listen signal at 5283.64 MHz obtained from scanning the phase of the second pulse of the sequence given in Supplementary Figure 7 (4373 MHz).

```
# Phase of the listen signal, degrees
# Column #1 -- phase of the scanned pulse, in degrees
# Column #2 -- mean phase for all measurements
# Column #3 -- standard deviation of the phase for all measurements
# Columns from #4 and on -- the individually measured points
  0   161.1     5.1    158.5   157.8   154.9   167.8   166.4
 18   135.3     8.4    129.3   143.1   129.1   127.5   147.7
 36   109.2     3.8    114.1   106.9   104.2   111.4
 54    97.6     4.8     99.4   105.7    96.2    91.6    95.1
 72    87.9    84.3     85.5    93.8    91.6    86.7    82.0
 90    83.1    67.5     81.6    92.0    79.1    81.5    81.1
108    79.4     4.3     72.6    76.2    83.9    82.5    82.0
126    70.5     4.0     71.8    66.9    65.1    76.3    72.7
144    64.2     4.9     62.0    56.2    65.6    66.7    70.6
162    52.3     3.2     52.0    48.4    56.2    55.7    49.4
180    39.3     5.1     38.6    32.3    36.0    47.3    42.2
```

198	28.9	1.7	27.3	30.5	26.5	30.7	29.3
216	21.7	7.6	33.6	24.5	16.6	11.2	22.8
234	19.1	5.2	13.2	17.2	24.3	26.0	14.5
252	17.3	4.8	13.9	24.1	14.6	22.0	12.1
270	20.9	7.2	14.7	31.2	13.2	27.8	17.6
288	29.1	31.6	45.2	41.4	1.3	72.2	-14.9
306	32.9	10.8	-168.2	-173.0	164.0	173.0	168.5
324	100.0	8.3	178.1	160.6	-176.3	165.9	171.5
342	169.8	4.7	160.5	171.4	172.9	171.1	173.1
360	155.9	5.6	146.8	163.6	153.5	158.4	157.3

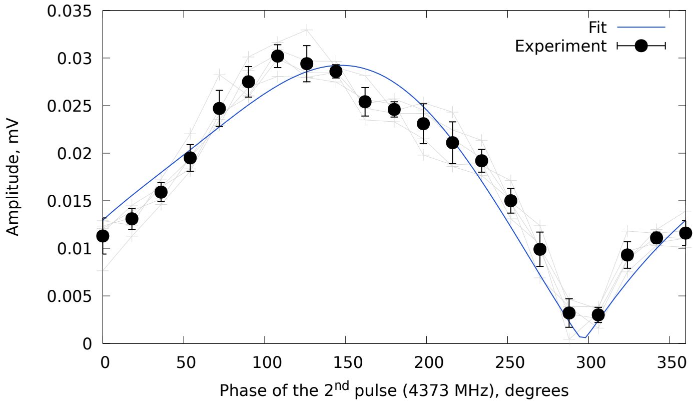

Supplementary Figure 23: The experimental amplitude of the listen signal at 5283.64 MHz obtained from scanning the phase of the second pulse of the sequence given in Supplementary Figure 7 (4373 MHz) and its fit. The circles with error bars are the values averaged over the individual measurements (given as gray lines with cross-shaped points). Solid blue line represents the fit result.

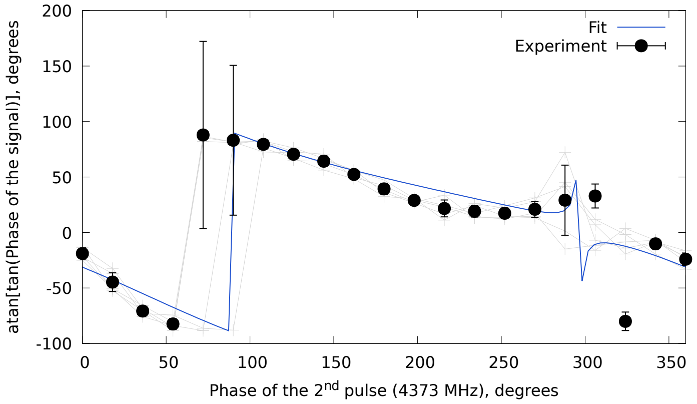

Supplementary Figure 24: The experimental phase of the listen signal at 5283.64 MHz obtained from scanning the phase of the second pulse of the sequence given in Supplementary Figure 7 (4373 MHz) and its fit. The circles with error bars are the values averaged over the individual measurements (given as gray lines with cross-shaped points). Solid blue line represents the fit result.

Supplementary Table 7: Fitted parameters of the listen signal at 5283.64 MHz from scanning the phase of the second pulse of the sequence given in Supplementary Figure 7 (4373 MHz).

Signal no.	Signature (s)	Amplitude (A, mV)	Phase (ϕ, degrees)
1	-1	0.0139271202	21.9534498820
2	0	0.0141606079	-103.9489540975
3	1	0.0024113986	49.0831151645

4.3 Phase scan for pulse #3 (5293 MHz)

Supplementary Table 8: Amplitude (in mV) of the listen signal at 5283.64 MHz obtained from scanning the phase of the third pulse of the sequence given in Supplementary Figure 7 (5293 MHz).

```
# Intensity of the listen signal, mV
# Column #1 -- phase of the scanned pulse, in degrees
# Column #2 -- mean intensity for all measurements
# Column #3 -- standard deviation of the intensity for all measurements
# Columns from #4 and on -- the individually measured points
   0 0.0099 0.0012  0.0095 0.0085 0.0113 0.0114 0.0090
  18 0.0172 0.0015  0.0157 0.0168 0.0192 0.0188 0.0155
  36 0.0210 0.0012  0.0203 0.0197 0.0202 0.0226 0.0223
  54 0.0250 0.0011  0.0240 0.0270 0.0251 0.0249 0.0242
  72 0.0222 0.0010  0.0234 0.0225 0.0230 0.0212 0.0208
  90 0.0204 0.0021  0.0206 0.0235 0.0216 0.0173 0.0190
 108 0.0148 0.0007  0.0146 0.0150 0.0155 0.0136 0.0154
 126 0.0081 0.0005  0.0083 0.0084 0.0078 0.0073 0.0087
 144 0.0020 0.0011  0.0034 0.0029 0.0014 0.0004 0.0018
 162 0.0082 0.0012  0.0077 0.0080 0.0102 0.0085 0.0065
 180 0.0157 0.0019  0.0137 0.0167 0.0170 0.0178 0.0130
 198 0.0212 0.0013  0.0221 0.0218 0.0218 0.0220 0.0186
 216 0.0263 0.0021  0.0252 0.0260 0.0302 0.0258 0.0241
 234 0.0289 0.0019  0.0306 0.0297 0.0295 0.0298 0.0252
 252 0.0287 0.0009  0.0298 0.0297 0.0277 0.0282 0.0283
 270 0.0254 0.0017  0.0272 0.0246 0.0257 0.0227 0.0269
 288 0.0206 0.0010  0.0195 0.0212 0.0219 0.0195 0.0212
 306 0.0138 0.0012  0.0147 0.0119 0.0145 0.0129 0.0149
 324 0.0061 0.0019  0.0082 0.0030 0.0056 0.0078 0.0058
 342 0.0048 0.0009  0.0045 0.0038 0.0049 0.0066 0.0044
 360 0.0104 0.0010  0.0095 0.0112 0.0105 0.0117 0.0089
```

Supplementary Table 9: Phase (in degrees) of the listen signal at 5283.64 MHz obtained from scanning the phase of the third pulse of the sequence given in Supplementary Figure 7 (5293 MHz).

```
# Phase of the listen signal, degrees
# Column #1 -- phase of the scanned pulse, in degrees
# Column #2 -- mean phase for all measurements
# Column #3 -- standard deviation of the phase for all measurements
# Columns from #4 and on -- the individually measured points
  0   136.3    7.4    135.5   127.2   130.1   140.9   147.7
 18   137.9    5.4    138.6   136.1   138.6   129.5   146.4
 36   134.7    5.4    125.3   139.9   133.0   135.5   140.1
 54   135.3    5.5    129.1   129.2   139.6   135.6   142.9
 72   133.4    2.8    133.4   136.6   136.4   129.9   130.9
 90   133.8    4.7    131.4   138.4   127.5   140.1   131.7
108   131.7    2.6    129.0   136.2   133.0   130.8   129.7
126   138.2    8.5    125.4   134.7   151.3   141.8   137.9
144   -12.1   31.6    167.6  -101.3  -149.1   179.1  -157.1
162   -72.3    7.7    -70.2   -69.6   -84.1   -76.4   -61.0
180   -58.6   11.6    -76.0   -57.9   -63.3   -55.4   -40.4
198   -55.6    2.5    -52.9   -58.4   -52.7   -58.5   -55.2
216   -55.1    6.7    -56.9   -66.5   -54.0   -51.9   -46.3
234   -54.1    6.1    -55.7   -55.2   -58.2   -59.2   -42.4
252   -56.6    3.6    -60.1   -56.4   -60.5   -50.5   -55.6
270   -59.8    4.0    -64.5   -59.2   -63.7   -57.9   -53.5
288   -61.4    4.5    -67.3   -62.9   -54.8   -64.3   -57.8
306   -62.2    7.1    -71.8   -63.2   -57.4   -67.0   -51.5
324   -75.9   55.4    -71.7   -55.0   -71.4  -109.9   -71.6
342   -30.6   33.0   -126.4   142.0   158.5  -175.2  -152.1
360   137.0    6.6    140.3   138.6   125.3   145.2   135.7
```

Supplementary Table 10: Fitted parameters of the listen signal at 5283.64 MHz from scanning the phase of the third pulse of the sequence given in Supplementary Figure 7 (5293 MHz).

Signal no.	Signature (s)	Amplitude (A, mV)	Phase (ϕ, degrees)
1	0	0.0032746074	87.9434872221
2	-1	0.0129164048	2.9322341773
3	1	0.0135882941	-116.5492307702

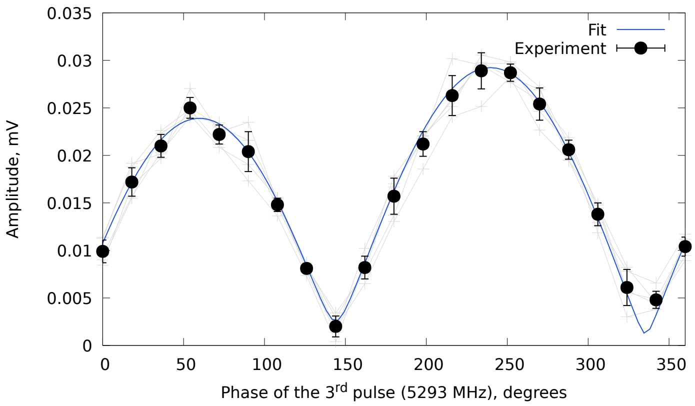

Supplementary Figure 25: The experimental amplitude of the listen signal at 5283.64 MHz obtained from scanning the phase of the third pulse of the sequence given in Supplementary Figure 7 (5293 MHz) and its fit. The circles with error bars are the values averaged over the individual measurements (given as gray lines with cross-shaped points). Solid blue line represents the fit result.

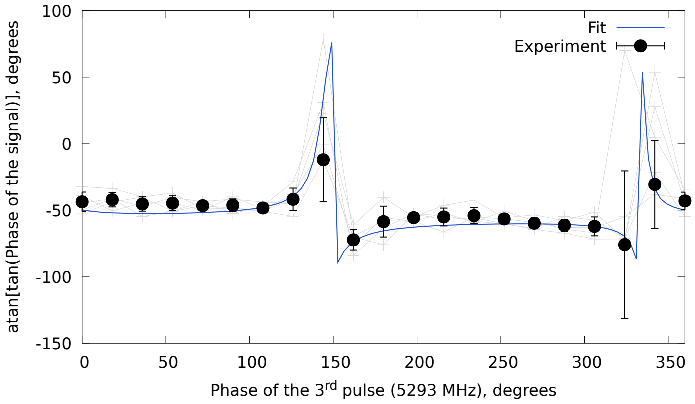

Supplementary Figure 26: The experimental phase of the listen signal at 5283.64 MHz obtained from scanning the phase of the third pulse of the sequence given in Supplementary Figure 7 (5293 MHz) and its fit. The circles with error bars are the values averaged over the individual measurements (given as gray lines with cross-shaped points). Solid blue line represents the fit result.

4.4 Phase scan for pulse #4 (9231 MHz)

Supplementary Table 11: Amplitude (in mV) of the listen signal at 5283.64 MHz obtained from scanning the phase of the fourth pulse of the sequence given in Supplementary Figure 7 (9231 MHz).

```
# Intensity of the listen signal, mV
# Column #1 -- phase of the scanned pulse, in degrees
# Column #2 -- mean intensity for all measurements
# Column #3 -- standard deviation of the intensity for all measurements
# Columns from #4 and on -- the individually measured points
   0 0.0265 0.0015  0.0284 0.0281 0.0261 0.0242 0.0260
  18 0.0220 0.0010  0.0231 0.0227 0.0203 0.0221 0.0216
  36 0.0208 0.0002  0.0209 0.0204 0.0209 0.0208 0.0209
  54 0.0215 0.0006  0.0227 0.0209 0.0215 0.0212 0.0211
```

```
 72 0.0168 0.0011  0.0169 0.0159 0.0186 0.0170 0.0157
 90 0.0129 0.0007  0.0139 0.0119 0.0133 0.0128 0.0125
108 0.0121 0.0010  0.0118 0.0128 0.0111 0.0137 0.0112
126 0.0075 0.0016  0.0048 0.0099 0.0073 0.0079 0.0078
144 0.0091 0.0018  0.0101 0.0100 0.0080 0.0063 0.0112
162 0.0065 0.0006  0.0064 0.0072 0.0062 0.0055 0.0070
180 0.0029 0.0014  0.0025 0.0029 0.0055 0.0018 0.0014
198 0.0060 0.0006  0.0071 0.0059 0.0056 0.0060 0.0055
216 0.0075 0.0010  0.0061 0.0069 0.0083 0.0088 0.0075
234 0.0116 0.0010  0.0124 0.0108 0.0120 0.0127 0.0101
252 0.0143 0.0014  0.0154 0.0138 0.0155 0.0148 0.0118
270 0.0181 0.0013  0.0174 0.0195 0.0160 0.0186 0.0192
288 0.0229 0.0012  0.0226 0.0230 0.0214 0.0250 0.0227
306 0.0243 0.0006  0.0243 0.0249 0.0235 0.0251 0.0240
324 0.0238 0.0015  0.0243 0.0259 0.0231 0.0244 0.0213
342 0.0266 0.0017  0.0262 0.0299 0.0258 0.0261 0.0251
360 0.0233 0.0013  0.0248 0.0223 0.0240 0.0241 0.0213
```

Supplementary Table 12: Phase (in degrees) of the listen signal at 5283.64 MHz obtained from scanning the phase of the fourth pulse of the sequence given in Supplementary Figure 7 (9231 MHz).

```
# Phase of the listen signal, degrees
# Column #1 -- phase of the scanned pulse, in degrees
# Column #2 -- mean phase for all measurements
# Column #3 -- standard deviation of the phase for all measurements
# Columns from #4 and on -- the individually measured points
   0    65.0     7.1     59.2    74.5    56.7    62.4    71.9
  18    81.2     7.5     66.9    83.6    85.5    88.6    81.2
  36    85.1     3.4     89.4    79.0    85.1    86.3    85.9
  54    93.0    69.5     88.0    91.8    92.3    99.1    93.8
  72   110.3     6.1    105.4   113.6   100.9   116.7   114.8
  90   115.0     4.2    112.0   111.3   115.6   122.9   113.5
 108   131.0     4.0    135.9   128.8   127.1   127.4   135.8
 126   134.8     7.5    129.7   130.8   149.5   134.5   129.7
 144   159.0     6.0    158.8   154.9   157.9   153.0   170.1
 162    20.8    17.2    148.8   146.8  -176.8   157.9  -172.7
 180   -36.2    14.9    -21.9   -23.9   -28.8   -45.7   -60.7
```

198	-11.8	20.3	-30.6	27.3	-16.1	-15.8	-23.7
216	-8.6	8.2	-12.2	5.7	-18.4	-5.9	-12.4
234	5.7	4.7	7.0	6.8	7.7	10.3	-3.5
252	20.1	9.5	26.1	16.4	35.1	15.0	8.1
270	35.0	3.7	31.2	35.7	36.4	30.9	40.9
288	45.6	6.3	43.2	46.2	44.9	37.0	56.5
306	53.7	5.6	49.6	64.0	53.5	53.6	47.8
324	64.0	4.2	64.7	69.4	57.3	61.6	66.9
342	66.4	5.6	58.6	70.3	60.7	72.4	70.0
360	69.8	4.9	64.5	72.7	63.4	76.0	72.2

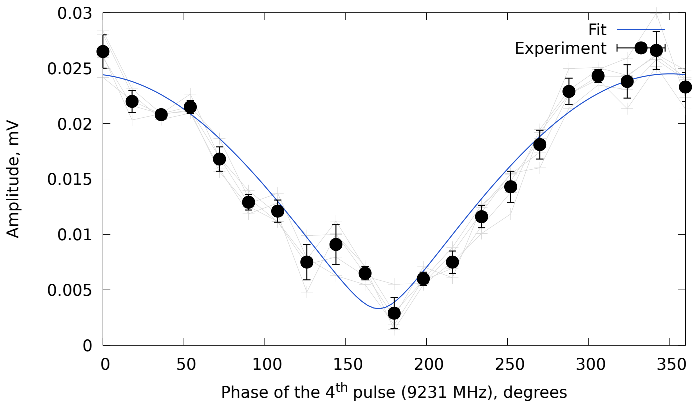

Supplementary Figure 27: The experimental amplitude of the listen signal at 5283.64 MHz obtained from scanning the phase of the fourth pulse of the sequence given in Supplementary Figure 7 (9231 MHz) and its fit. The circles with error bars are the values averaged over the individual measurements (given as gray lines with cross-shaped points). Solid blue line represents the fit result.

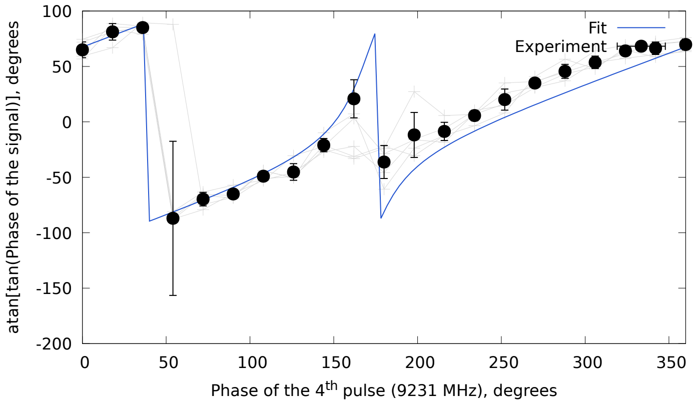

Supplementary Figure 28: The experimental phase of the listen signal at 5283.64 MHz obtained from scanning the phase of the fourth pulse of the sequence given in Supplementary Figure 7 (9231 MHz), and its fit. The circles with error bars are the values averaged over the individual measurements (given as gray lines with cross-shaped points). Solid blue line represents the fit result.

Supplementary Table 13: Fitted parameters of the listen signal at 5283.64 MHz from scanning the phase of the fourth pulse of the sequence given in Supplementary Figure 7 (9231 MHz).

Signal no.	Signature (s)	Amplitude (A, mV)	Phase (ϕ, degrees)
1	0	0.0105922321	62.0629994578
2	1	0.0138957432	71.7434785533

4.5 Phase scan for pulse #5 (3459 MHz)

Supplementary Table 14: Amplitude (in mV) of the listen signal at 5283.64 MHz obtained from scanning the phase of the fifth pulse of the sequence given in Supplementary Figure 7 (3459 MHz).

```
# Intensity of the listen signal, mV
# Column #1 -- phase of the scanned pulse, in degrees
# Column #2 -- mean intensity for all measurements
# Column #3 -- standard deviation of the intensity for all measurements
# Columns from #4 and on -- the individually measured points
  0 0.0101 0.0016  0.0119 0.0091 0.0121 0.0113 0.0083 0.0067 0.0103 0.0105 0.0105
 18 0.0133 0.0022  0.0153 0.0161 0.0151 0.0149 0.0122 0.0091 0.0124 0.0109 0.0139
 36 0.0170 0.0020  0.0182 0.0184 0.0164 0.0193 0.0154 0.0155 0.0128 0.0184 0.0186
 54 0.0178 0.0006  0.0176 0.0172 0.0177 0.0174 0.0182 0.0192 0.0184 0.0172 0.0172
 72 0.0192 0.0011  0.0198 0.0213 0.0195 0.0193 0.0185 0.0202 0.0187 0.0178 0.0178
 90 0.0196 0.0006  0.0200 0.0196 0.0187 0.0204 0.0199 0.0196 0.0195 0.0202 0.0183
108 0.0192 0.0014  0.0180 0.0207 0.0167 0.0185 0.0196 0.0210 0.0187 0.0208 0.0189
126 0.0193 0.0010  0.0193 0.0180 0.0204 0.0201 0.0185 0.0201 0.0199 0.0200 0.0177
144 0.0189 0.0016  0.0169 0.0168 0.0172 0.0195 0.0208 0.0182 0.0213 0.0199 0.0199
162 0.0183 0.0014  0.0186 0.0175 0.0185 0.0152 0.0199 0.0200 0.0188 0.0177 0.0181
180 0.0171 0.0017  0.0148 0.0158 0.0177 0.0144 0.0192 0.0171 0.0185 0.0192 0.0170
198 0.0151 0.0016  0.0141 0.0137 0.0137 0.0132 0.0153 0.0174 0.0181 0.0146 0.0162
216 0.0155 0.0019  0.0130 0.0136 0.0169 0.0140 0.0185 0.0141 0.0179 0.0153 0.0164
234 0.0126 0.0015  0.0111 0.0107 0.0113 0.0115 0.0149 0.0131 0.0147 0.0118 0.0141
252 0.0086 0.0020  0.0075 0.0051 0.0065 0.0093 0.0107 0.0107 0.0110 0.0092 0.0074
270 0.0088 0.0013  0.0084 0.0090 0.0107 0.0093 0.0097 0.0099 0.0084 0.0084 0.0056
288 0.0069 0.0016  0.0069 0.0082 0.0096 0.0087 0.0068 0.0066 0.0064 0.0054 0.0038
306 0.0051 0.0012  0.0055 0.0059 0.0060 0.0064 0.0065 0.0035 0.0031 0.0037 0.0050
324 0.0070 0.0013  0.0077 0.0082 0.0084 0.0082 0.0059 0.0054 0.0064 0.0047 0.0081
342 0.0088 0.0014  0.0097 0.0092 0.0112 0.0081 0.0087 0.0100 0.0083 0.0074 0.0061
360 0.0093 0.0016  0.0120 0.0110 0.0101 0.0090 0.0097 0.0080 0.0071 0.0071 0.0100
```

Supplementary Table 15: Phase (in degrees) of the listen signal at 5283.64 MHz obtained from scanning the phase of the fifth pulse of the sequence given in Supplementary Figure 7 (3459 MHz).

```
# Phase of the listen signal, degrees
# Column #1 -- phase of the scanned pulse, in degrees
# Column #2 -- mean phase for all measurements
# Column #3 -- standard deviation of the phase for all measurements
# Columns from #4 and on -- the individually measured points
  0  160.4   8.9   155.6  168.2  175.7  149.9  160.0  153.1  170.4  149.3  161.7
 18  137.7   8.3   143.4  140.2  133.3  147.0  140.1  143.4  135.7  117.0  139.4
 36  132.3   7.2   128.7  124.2  131.3  141.6  138.4  145.3  128.2  124.7  127.9
 54  121.2   3.7   122.9  122.6  120.8  127.4  125.5  118.8  120.6  114.7  117.4
 72  110.1   5.3   109.2  121.0  105.1  110.6  108.1  106.5  104.4  107.9  117.7
 90   99.1   3.7    96.0  101.1   98.5  105.0   98.6   99.0   92.2  103.9   97.2
108   87.2  84.1    86.4   78.5   94.8   84.2   79.5   83.6   91.9   90.0   96.1
126   75.0   4.8    74.5   76.4   82.2   82.2   73.3   70.3   76.7   73.1   66.4
144   60.5   4.5    65.1   63.3   60.0   62.9   57.8   57.1   61.4   50.8   66.1
162   49.9   7.0    38.5   55.1   55.3   50.1   56.7   60.1   46.5   41.9   44.5
180   36.9   8.7    46.4   29.9   29.4   19.8   41.4   45.1   44.7   42.1   32.9
198   18.4   9.3     3.2    7.8   16.0    9.9   26.1   20.1   24.3   32.7   25.6
216   13.7   5.8    13.5   10.2    7.2   13.2   10.6    7.3   26.4   14.9   19.8
234   78.9   7.1     2.4    9.1  -13.3   -5.8    1.6    0.8    7.2  349.7  358.3
252  139.6  13.1   -21.8  -45.7  -35.4  -27.4  -16.0  350.1  353.2  342.4  357.3
270  109.2  15.4   -61.6  -55.6  -78.2  -61.4  -41.5  324.8  326.1  330.3  300.2
288   86.1  63.0   -79.9 -102.5  -71.6  -90.8  -68.0  298.3  300.8  297.1  291.3
306   47.5  46.3  -111.9 -127.6 -119.6 -110.1 -112.6  267.6  210.6  247.7  283.2
324    5.0  11.5  -173.0 -164.4 -159.4 -151.8 -148.4  210.7  192.3  226.0  213.3
342   24.1   8.5  -177.6 -179.7 -177.7 -161.9  178.6  200.8  181.1  178.0  175.2
360  157.7  10.2   142.5  148.8  172.1  152.6  145.7  166.7  169.6  161.6  159.8
```

Supplementary Table 16: Fitted parameters of the listen signal at 5283.64 MHz from scanning the phase of the fifth pulse of the sequence given in Supplementary Figure 7 (3459 MHz).

Signal no.	Signature (s)	Amplitude (A, mV)	Phase (ϕ, degrees)
1	-1	0.0129253134	-168.2969572464
2	0	0.0063195620	80.6540822744

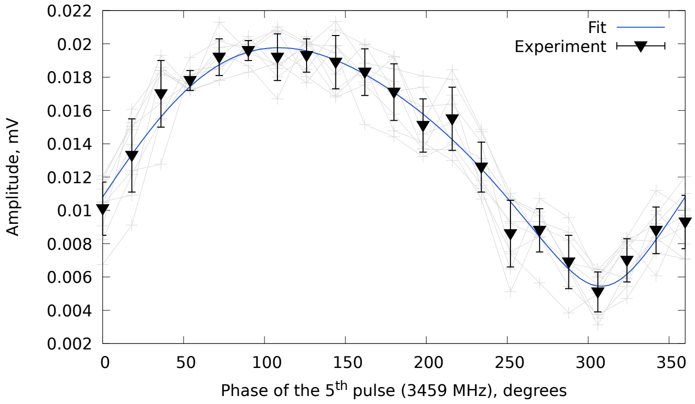

Supplementary Figure 29: The experimental amplitude of the listen signal at 5283.64 MHz obtained from scanning the phase of the fifth pulse of the sequence given in Supplementary Figure 7 (3459 MHz) and its fit. The circles with error bars are the values averaged over the individual measurements (given as gray lines with cross-shaped points). Solid blue line represents the fit result.

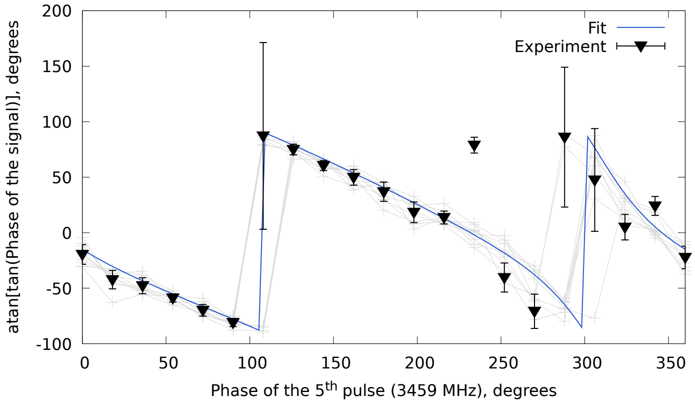

Supplementary Figure 30: The experimental phase of the listen signal at 5283.64 MHz obtained from scanning the phase of the fifth pulse of the sequence given in Supplementary Figure 7 (3459 MHz) and its fit. The circles with error bars are the values averaged over the individual measurements (given as gray lines with cross-shaped points). Solid blue line represents the fit result.

Supplementary references

(1) Rabi, I. I. Space quantization in a gyrating magnetic field. *Phys. Rev.* **51**, 652–654 (1937).

(2) Landau, L. D., Lifshitz, L. M. *Quantum Mechanics Non-Relativistic Theory, Third Edition: Volume 3* (Butterworth-Heinemann, 1981).

(3) Bychkov, S. S., Grishanin, B. A., Zadkov, V. N. Laser synthesis of chiral molecules in isotropic racemic media. *J. Exp. Theor. Phys.* **93**, 24–32 (2001).

(4) Bychkov, S. S., Grishanin, B. A., Zadkov, V. N., Takahashi, H. Laser coherent control of molecular chiral states via entanglement of the rotational and torsional degrees of freedom. *J. Raman Spectrosc.* **33**, 962–973 (2002).

(5) Zhdanov, D. V., Zadkov, V. N. Absolute asymmetric synthesis from an isotropic racemic mixture of chiral molecules with the help of their laser orientation-dependent selection. *J. Chem. Phys.* **127**, 244312 (2007).

(6) Quack, M., Seyfang, G. In *Molecular Spectroscopy and Quantum Dynamics*; Marquardt, R., Quack, M., Eds.; pp 231–282 (Elsevier, 2021).

(7) IUPAC, IUPAC Compendium of Chemical Terminology – The Gold Book. `http://goldbook.iupac.org/` (2009).

(8) Becke, A. D. Density-functional thermochemistry. III. The role of exact exchange. *J. Chem. Phys.* **98**, 5648–5652 (1993).

(9) Lee, C., Yang, W., Parr, R. G. Development of the Colle-Salvetti correlation-energy formula into a functional of the electron density. *Phys. Rev. B* **37**, 785–789 (1988).

(10) Vosko, S. H., Wilk, L., Nusair, M. Accurate spin-dependent electron liquid correlation energies for local spin density calculations: a critical analysis. *Canadian Journal of Physics* **58**, 1200–1211 (1980).

(11) Grimme, S., Ehrlich, S., Goerigk, L. Effect of the damping function in dispersion corrected density functional theory. *J. Comput. Chem.* **32**, 1456–1465 (2011).

(12) Weigend, F., Ahlrichs, R. Balanced basis sets of split valence, triple zeta valence and quadruple zeta valence quality for H to Rn: Design and assessment of accuracy. *Phys. Chem. Chem. Phys.* **7**, 3297–3305 (2005).

(13) Neese, F., Wennmohs, F., Becker, U., Riplinger, C. The ORCA quantum chemistry program package. *J. Chem. Phys.* **152**, 224108 (2020).

(14) Bataev, V., Abramenkov, A., Godunov, I. Complex character of internal rotation of furfural in the ground S_0 and excited S_1 electronic states. *J. Quant. Spectrosc. Radiat. Transf.* **255**, 107205, (2020).

(15) Godunov, I. A., Terentiev, R. V., Maslov, D. V., Yakovlev, N. N., Bataev, V. A., Abramenkov, A. V. The structure of 2-methylpropanal molecule in the S_1 lowest excited singlet electronic state: theoretical and experimental studies. *Struct. Chem.* **30**, 529–544, (2019).

(16) Bokarev, S., Pupyshev, V., Godunov, I. Vibronic spectra, ab initio calculations, and structures of conformationally non-rigid molecules of oxalyl halides in the ground and lowest excited electronic states. Part II: Theoretical investigation of oxalyl chloride. *J. Mol. Spectrosc.* **256**, 247–255, (2009).

(17) Kudich, A., Bataev, V., Abramenkov, A., Pupyshev, V., Godunov, I. Theoretical study of the structure of propanal in the first excited singlet and triplet electronic states: three-dimensional model for torsional and inversion vibrations. *J. Mol. Struct.: THEOCHEM* **631**, 39–51, (2003).

(18) Fatima, M., Maué, D., Pérez, C., Tikhonov, D. S., Bernhard, D., Stamm, A., Medcraft, C., Gerhards, M., Schnell, M. Structures and internal dynamics of diphenylether and its aggregates with water. *Phys. Chem. Chem. Phys.* **2020**, *22*, 27966–27978 (2020).

(19) Utzat, K. A., Bohn, R. K., Montgomery, J. A., Michels, H. H., Caminati, W. Rotational spectrum, tunneling motions, and potential barriers of benzyl alcohol. *J. Phys. Chem. A* **114**, 6913–6916 (2010).

(20) Evangelisti, L., Caminati, W. Modeling the internal rotation tunnelling in benzyl alcohol by ring fluorination: The rotational spectrum of 3,5-difluorobenzyl alcohol. *Chem. Phys. Lett.: X* **1**, 100004 (2019).

(21) Schmitz, D., Alvin Shubert, V., Betz, T., Schnell, M. Multi-resonance effects within a single chirp in broadband rotational spectroscopy: The rapid adiabatic passage regime for benzonitrile. *J. Mol. Spectrosc.* **280**, 77–84, Broadband Rotational Spectroscopy (2012).

(22) Pérez, C., Steber, A. L., Krin, A., Schnell, M. State-Specific Enrichment of Chiral Conformers with Microwave Spectroscopy. *J. Phys. Chem. Lett.* **9**, 4539–4543 (2018).

(23) Glauber, R. J. Photon correlations. *Phys. Rev. Lett.* **10**, 84 (1963).

(24) Leibscher, M., Pozzoli, E., Pérez, C., Schnell, M., Sigalotti, M., Boscain, U., Koch, C. P. Full quantum control of enantiomer-selective state transfer in chiral molecules despite degeneracy. *Comm. Phys.* **5**, 110 (2022).

(25) Press, W. H., Teukolsky, S. A., Vetterling, W. T., Flannery, B. P. *Numerical Recipes 3rd Edition: The Art of Scientific Computing*, 3rd ed. (Cambridge University Press, 2007).

(26) Milton Abramowitz, I. A. S. *Handbook of Mathematical Functions: with Formulas, Graphs, and Mathematical Tables*, 10th ed.; Applied Mathematics Series 55 (NBS, 1972).
